# Optimization of photon counting CT for cardiac imaging in patients with left ventricular assist devices: An in‐depth assessment of metal artifacts

**DOI:** 10.1002/acm2.14386

**Published:** 2024-05-13

**Authors:** Bente Konst, Linus Ohlsson, Lilian Henriksson, Mårten Sandstedt, Anders Persson, Tino Ebbers

**Affiliations:** ^1^ Department of Health, Medicine and Caring Sciences Linköping University Linköping Sweden; ^2^ Center for Medical Image Science and Visualization (CMIV) Linköping University Linköping Sweden; ^3^ Department of Radiology Vestfold Hospital Tønsberg Norway; ^4^ Department of Thoracic and Vascular Surgery in Östergötland, and Department of Health Medicine and Caring Sciences Linköping University Linköping Sweden; ^5^ Department of Radiology in Linköping and Department of Health Medicine and Caring Sciences Linköping University Linköping Sweden

**Keywords:** heart, left ventricular assistance device, metal artifact, myocardium, photon‐counting computed tomography

## Abstract

**Purpose:**

Photon counting CT (PCCT) holds promise for mitigating metal artifacts and can produce virtual mono‐energetic images (VMI), while maintaining temporal resolution, making it a valuable tool for characterizing the heart. This study aimed to evaluate and optimize PCCT for cardiac imaging in patients during left ventricular assistance device (LVAD) therapy by conducting an in‐depth objective assessment of metal artifacts and visual grading.

**Methods:**

Various scan and reconstruction settings were tested on a phantom and further evaluated on a patient acquisition to identify the optimal protocol settings. The phantom comprised an empty thoracic cavity, supplemented with heart and lungs from a cadaveric lamb. The heart was implanted with an LVAD (HeartMate 3) and iodine contrast. Scans were performed on a PCCT (NAEOTOM Alpha, Siemens Healthcare). Metal artifacts were assessed by three objective methods: Hounsfield units (HU)/SD measurements (Diff_HU_ and SD_ARTIFACT_), Fourier analysis (AmplitudeLowFreq), and depicted LVAD volume in the images (BloomVol). Radiologists graded metal artifacts and the diagnostic interpretability in the LVAD lumen, cardiac tissue, lung tissue, and spinal cord using a 5‐point rating scale. Regression and correlation analysis were conducted to determine the assessment method most closely associated with acquisition and reconstruction parameters, as well as the objective method demonstrating the highest correlation with visual grading.

**Results:**

Due to blooming artifacts, the LVAD volume fluctuated between 27.0 and 92.7 cm^3^. This variance was primarily influenced by kVp, kernel, keV, and iMAR (*R* ^2 ^= 0.989). Radiologists favored pacemaker iMAR, 3 mm slice thickness, and T3D keV and kernel Bv56f for minimal metal artifacts in cardiac tissue assessment, and 110 keV and Qr40f for lung tissue interpretation. The model adequacy for Diff_HU_ SD_ARTIFACT_, AmplitueLowFreq, and BloomVol was 0.28, 0.76, 0.29, and 0.99 respectively for phantom data, and 0.95, 0.98, 1.00, and 0.99 for in‐vivo data. For in‐vivo data, the correlation between visual grading (VG_SUM_) and Diff_HU_ SD_ARTIFACT_, AmplitueLowFreq, and BloomVol was −0.16, −0.01, −0.48, and −0.40 respectively.

**Conclusion:**

We found that optimal scan settings for LVAD imaging involved using 120 kVp and IQ level 80. Employing T3D with pacemaker iMAR, the sharpest allowed vascular kernel (Bv56f), and VMI at 110 keV with kernel Qr40 yields images suitable for cardiac imaging during LVAD‐therapy. Volumetric measurements of the LVAD for determination of the extent of blooming artifacts was shown to be the best objective method to assess metal artifacts.

## 1 INTRODUCTION

Heart failure is a global health concern, leading to millions of deaths annually.^1^, ^2^ In advanced stages, heart transplantation offers the best prognosis, but donor scarcity and contraindications limit this option.^3^ As a result, durable mechanical circulatory support (dMCS) has made important strides, mostly due to the technical development within left ventricular assist devices (LVADs).^4^ In the realm of LVAD therapy, the HeartMate 3 (HM3) (Abbott Laboratories, Lake Forest, IL), a fully magnetically levitated centrifugal flow pump, has seen increasing prominence in recent years, and its dominance is highlighted in the latest international MCS register reports.^5^ Patients suitable for LVAD therapy come with varied pathologies and the patient group demand individualized care and continuous optimization of system settings to prevent complications.^6^ While echocardiography is the gold standard to assess cardiac function during LVAD therapy, it faces notable challenges. The metal of the LVAD severely limits the acoustic window resulting in an inability to visualize the entire outflow graft^7^ and restricting assessment of thrombosis, bleeding, and infections.^8^ Time‐resolved computed tomography (CT) datasets allow the calculation of various functional cardiac parameters including volumes, ejection fraction, myocardial mass, and wall motion.^9^, ^10^ CT may also offer a valuable alternative to echocardiography for the detection of conditions such as thrombosis, cannula malposition, and bleeding, and its adoption has been on the rise.^11^ In energy integrated detector (EID) CT, the presence of metal in the LVAD commonly introduces image artifacts, such as beam hardening, blooming, noise, and scatter, which for instance explains the relatively low sensitivity and high specificity for detection of LVAD thrombus.^12^ These artifacts, combined with other prominent non‐optimal conditions in cardiac CT imaging, limit the diagnostic quality and the clinical utility.

Advantages of photon counting CT (PCCT) over EID include decreased electronic noise, improved spectral and spatial resolution, and increased contrast‐to‐noise ratio.^13^ LVAD patients often have renal dysfunction before LVAD implantation,^14^ hence it is favorable that PCCT allows for reduced iodine contrast media.^15^, ^16^ PCCT also holds promise for mitigating metal artifacts, particularly in the case of light metals.^17^, ^18^ PCCT can produce virtual mono‐energetic images (VMI), while maintaining temporal resolution, making it a valuable tool for characterizing the heart and myocardium.^15^, ^19^, ^20^, ^21^, ^22^

Three strategies to mitigate metal artifacts are: (1) simulation of mono‐energetic energies at high energy, (2) use of raw data with modified iterative reconstruction, (3) application of dedicated Metal Artefact Reduction (MAR) algorithms on projection data.^23^, ^24^, ^25^, ^26^

PCCT holds great promise for cardiac imaging in patients during LVAD therapy, and there is a consensus on the utilization of CT for postoperative imaging of LVAD patients.^11^, ^27^ However, a CT protocol delineating the optimal combination of metal artifact mitigating strategies for EID^11^ and PCCT for LVAD imaging is currently lacking. Very few studies describe CT in LVAD,^28^ and our study may provide knowledge about LVAD diagnostics with new technology.

This study aimed to evaluate and optimize PCCT for cardiac imaging in patients during LVAD therapy by conducting an in‐depth objective assessment of metal artifacts and visual grading. We assessed the impact of different scan and reconstruction parameter settings on image quality using a phantom. The outcomes of these phantom images guided the development of a CT protocol, which was then applied to an LVAD patient for further evaluation. Additionally, we explored potential correlations between visual grading and objective methods used to assess metal artifacts.

## 2 MATERIALS AND METHODS

**FIGURE 1:**
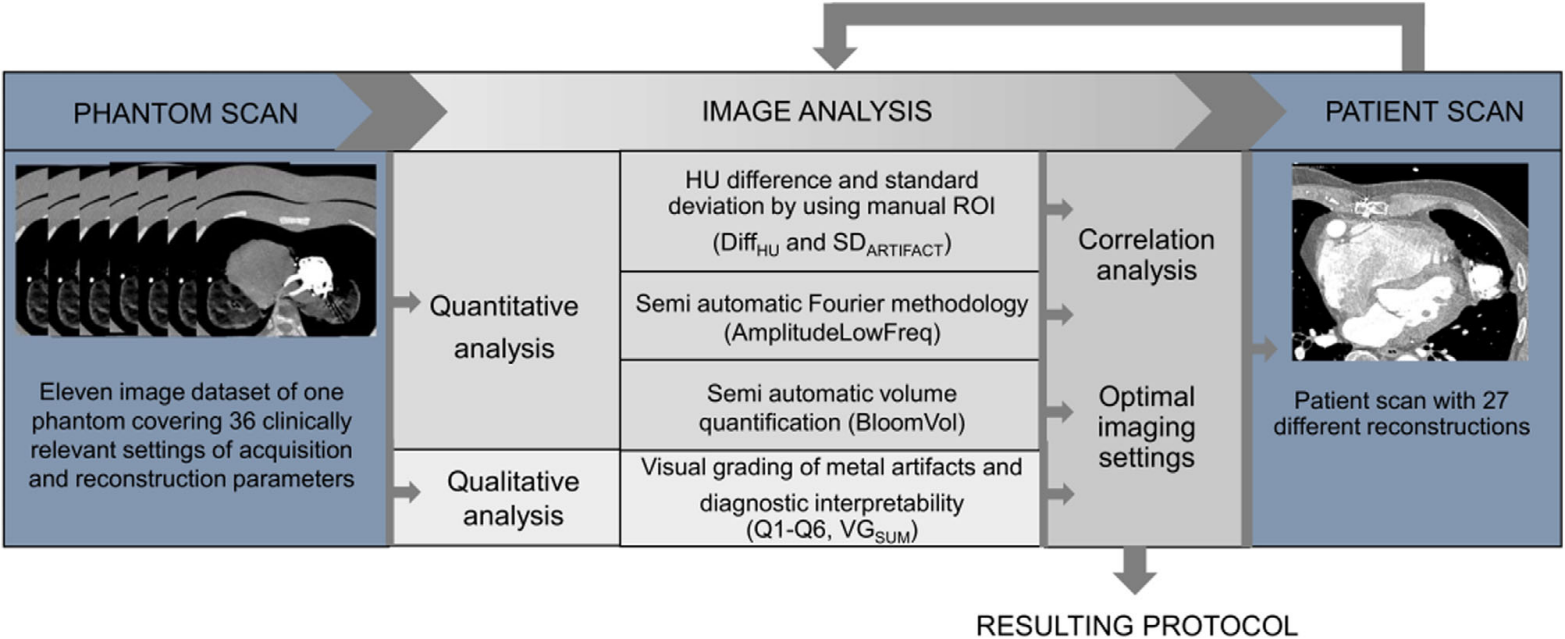
Flowchart of the study methodology.

Various scan and reconstruction settings were tested on a phantom and further evaluated in vivo. The phantom consisted of a commercial chest phantom withholding a lamb's heart and lungs, iodine contrast, and an LVAD. PCCT scans of the phantom were performed with various settings, including different kVp, dose levels, image filtration (kernels), slice thicknesses, and MAR presets. We determined the most suitable imaging parameters through a combination of visual grading and objective metal artifact assessment. To evaluate the correlation between metal artifact assessment methods and visual grading, we calculated Spearman's rho. Subsequently, we developed a patient protocol and obtained and evaluated an initial in‐vivo dataset within this study. For a detailed overview of our methodology, please refer to Figure 1, the study's flowchart.

### 2.1 Phantom and patient

**FIGURE 2:**
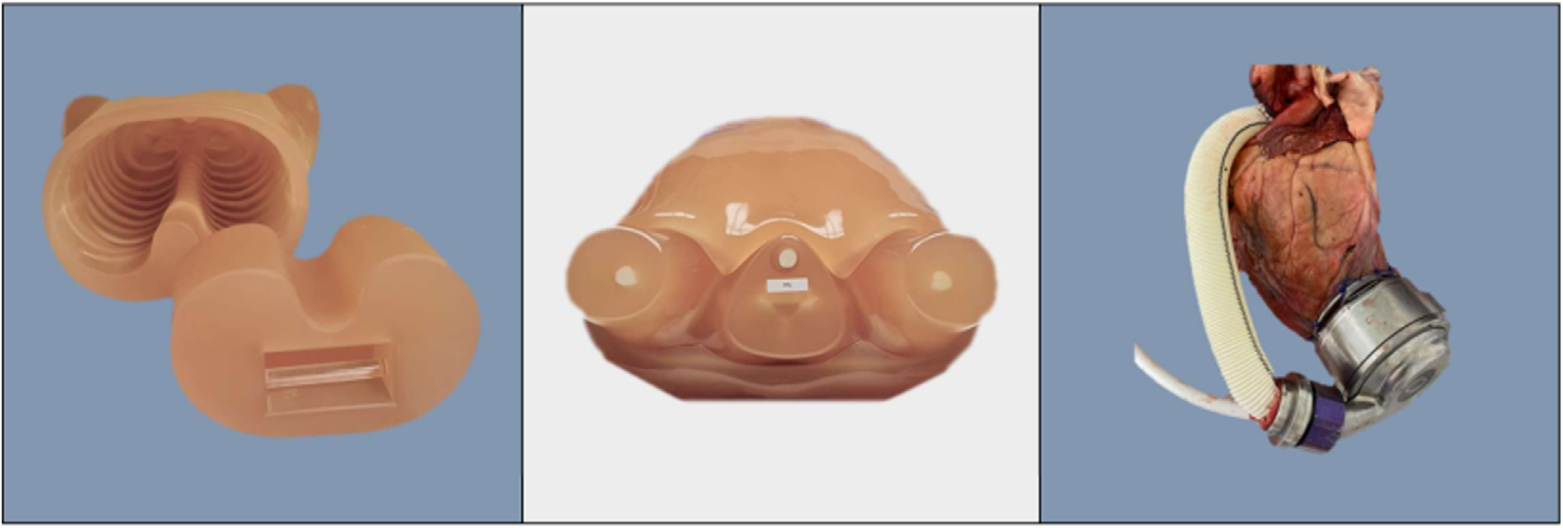
A multipurpose chest phantom N1, “Lungman,” PH‐1; Kyoto‐Kagaku. The lung insert was replaced with lamb heart and lungs with an LVAD. Extra chest plates were added to simulate a standard patient size.

The phantom utilized in this study was based on a commercially available chest phantom, supplemented with additional chest plates (Multipurpose Chest Phantom N1, Lungman, PH‐1; Kyoto‐Kagaku Co., Ltd., Kyoto, Japan).^29^ The phantom material of the inner thoracic cavity was replaced by a lamb's heart and lung, in which a real LVAD prototype (HeartMate 3), had been surgically sutured on to the left ventricular apex (see Figure 2). Additionally, the ventricles were filled with iodine contrast and simulated tumors 3.5 and 8 mm in diameter, with distinct nominal Hounsfield units (HU) values, were placed into the thoracic cavity. These tumors were crafted from urethane foam, with a nominal HU value of approximately −630. In contrast, polyurethane, SZ50, and hydroxyapatite displayed nominal HU values of +100.

The patient study was performed in agreement with the Declaration of Helsinki and had been approved by the Swedish Ethical Review Authority (Dnr: 2022‐06934‐01). The study participant provided informed written consent prior to participation. The inclusion criteria were patients associated with Linköping University hospital undergoing treatment with HeartMate 3 and aged 18 or older. Exclusion criteria included known iodine contrast allergies, reduced kidney function, pregnancy, newly diagnosed thyroid cancer, untreated hyperthyroidism and myasthenia gravis.

### 2.2 Image acquisition and reconstruction

Both phantom and patient underwent imaging using a dual‐source PCCT scanner (NAEOTOM Alpha, Siemens Healthcare, Forchheim, Germany). All scans were made using an electrocardiogram (ECG)‐triggered cardiac dual source spiral scan mode which enabled spectral post processing. The general CT scan parameters were set as follows: 0.25 s rotation time, 66 ms temporal resolution, 144 × 0.4 mm collimation, 0.31 pitch factor.

For the phantom scans, a synthetic ECG signal of 60 bpm was used. Automated tube current modulation (CARE Dose4D and CARE keV, Siemens) was employed, and the radiation dose was adjusted by changing the image quality (IQ) level. The kVp values were changed manually and the scans were optimized for vascular examinations. Preliminary phantom tests indicated that settings with 70 kVp did not yield satisfactory image quality. Neither did 90 kVp with an IQ level below 55. Various image reconstructions were made with different kernels, slice thicknesses, and iMAR (iterative metal artifact reconstruction, Siemens) settings. The spectral properties of the PCCT data were utilized to acquire VMI at different keV levels as well as poly‐energetic reconstructions, encompassing photon energies ranging from 20 to 120 keV (T3D). An energy threshold at 20 keV was automatically applied to all reconstructions for removal of electronic noise. Quantum iterative reconstruction (QIR) was set to a strength level of 4 for all images. All included scans and reconstructions are specified in Table 1.

**TABLE 1:** Detailed scan and reconstruction parameters for images acquired for both phantom and patient.

Tube Voltage (kVp)	Collimation (mm)	IQ	CTDI Vol (mGy)	Recon kernel	Slice thickness (mm)	VMI (keV)	iMAR preset
90	144 × 0.4	80	52.6	Qr40	0.4	110	Pacemaker
120			56.7			T3D	Pacemaker
140			58.8				
120	144 × 0.4	55	39	Qr40	0.4	110	Pacemaker
120		60	43.3				
120		80	56.9				
120		100	31.2				
140		55	39.6				
140		60	53.4				
140		80	58.8				
120	144 × 0.4	80	56.7	Qr36*	0.4	110	Pacemaker
				Qr40*			
				Qr44*			
				Bl56*			
				Bv36*			
				Bv40*			
				Bv44*			
				Bv56*			
				**Qr56** *			
120	144 × 0.4	80	56.9	Qr40/4*	0.4	110	None*
				Bv56**			Pacemaker*
							Thoracic Coils*
							Hip Implants*
							Extremity Implants*
120	144 × 0.4	80	56.9	Qr40	0.4	**T3D** *	Pacemaker
				Bv56**		40*	
						62*	
						SPP‐70 keV*	
						**90** *	
						110*	
						190*	
140	144 × 0.4	80	58.8	Qr40	0.4	T3D	Pacemaker
						40	
						67	
						110	
120	144 × 0.4	80	56.9	Qr40	0.4*	110	Pacemaker
				**Bv56** *	1.0*		
					3.0*		
140	144 × 0.4	80	58.8	Qr40	0.4	110	Pacemaker
					1.0		
					3.0		

*Note*: Bold text indicates reconstructions options new for the patient, and not performed for the phantom study. Virtual mono‐energetic images (VMI) from 40 to 190 keV and T3D that indicates poly‐energetic reconstruction, encompassing photon energies ranging from 20 to 120 keV.^13^

Abbreviations: Bl, body‐lung; Bv, body‐vascular, higher numeric values indicate increasing sharpness; CTDI, computed tomography dose index volume; IQ, image quality; Qr, quantitative regular; Recon kernel, Reconstruction kernel.

* and ** indicate reconstructions performed for the patient. ** indicates that Bv56 is only applied in combination with Thoracic Coils and Pacemaker preset/90 keV.

Based on the experiences gained from imaging the phantom, a patient protocol was established, which was tested on a patient with an LVAD. The acquisition was made using 120 kVp and IQ level 80. A biphasic contrast injection was used to ensure opacification of all four heart chambers. The first phase had a volume of 65 mL containing 80% iodine contrast (Omnipaque 350 mg I/mL GE Healthcare, Chicago IL, USA) and 20 % saline solution. This was followed by a second phase of 50 mL containing 30% of the same contrast agent and 70% saline solution. A saline chaser of 40 mL finished the injection which was made into an antecubital vein at 5 mL/s. Bolus tracking was used to achieve optimal contrast opacification with region of interest (ROI) placement in the descending aorta. The acquisition was initiated after reaching an increase of 100 HU with a 5 s delay to allow for breath hold instruction. Multiphase reconstructions were made that included reconstructions of the entire scan range at timepoints at every 5% of the cardiac cycle (20 phases in total). Only the best diastolic/systolic phases were included in this study for evaluation.

The resulting images were transferred to the in‐house Picture Archiving and Communication System (PACS). Image analyses were conducted on a standard PACS workstation (IDS7, Sectra Medical Systems GmbH, Linköping, Sweden) and subsequently loaded into ViewDex 3.0^30^ for visual grading. For quantitative analysis, Python 3.10 and ParaView 5.11.0 (kitware.com) were used.

### 2.3 Evaluation of images

A qualitative assessment was performed by six radiologists to determine the presence of metal artifacts and their diagnostic value in various tissues. Additionally, three different methods for the objective quantification of metal artifacts were employed.

#### 2.3.1 Qualitative evaluation: Absolute visual grading

The phantom image and subsequently patient images were showcased to the readers through ViewDex 3.0. This Java‐based software facilitates the presentation and evaluation of anonymized medical images in performance studies involving reviewers. Image cases were randomized by the software and each reader's responses were stored in a distinct log file.^30^, ^31^, ^32^, ^33^

**FIGURE 3:**
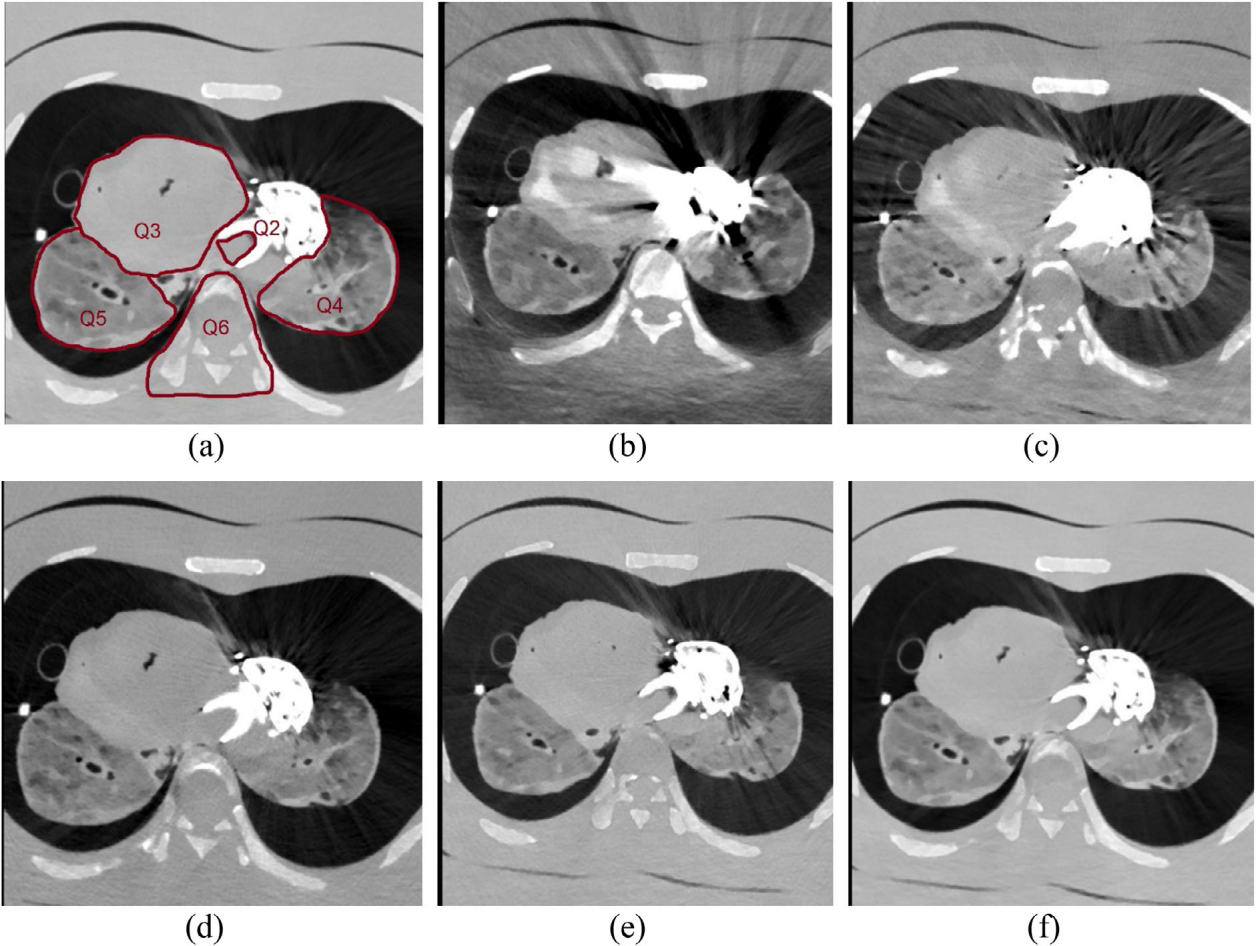
(a) Shows the areas where diagnostic interpretability was evaluated, Q2: in the lumen of the LVAD inflow cannula, Q3: the cardiac tissue, Q4: left lung tissue, Q5: right lung tissue and Q6: the spinal cord and surrounding tissue. (b–f), shows images with a variety of metal artifacts. (b) massive artefacts, (c) pronounced streaks, (d) minor streaks, (e) minor streaks only at the thickest portions of the metallic implant, (f) indicates the absence of artefacts.

Six independent readers assessed 36 phantom image sets and 27 patient image sets. Among them, four were experienced radiologists (20+ years of experience), one had 15 years of experience while the sixth was a junior medical doctor. They graded metal artifacts and their prominence in general (Q1). Since adjustments of imaging parameters may have various effects on image quality, due to different attenuation and tissue properties, the image evaluations were also conducted for different anatomic regions: in the lumen of the LVAD inflow cannula (Q2), cardiac tissue (Q3), lung tissue (Q4 and Q5), and the area around spinal cord (Q6), as shown in Figure 3a. All questions (Q1–Q6) were rated on an absolute 5‐point rating scale. During image review, the readers could adjust the window settings, zoom, and pan. All readers were familiar with the scale in Figure 3b–f before reviewing the images.

For scoring of general metal artifacts, Question 1 (Q1): a grade of 1 indicates massive artifacts, 2 indicates pronounced streaks, 3 indicates minor streaks, 4 represents minor streaks only at the thickest portions of the metallic implant, while a grade of 5 represents the absence of metal artifacts. In terms of diagnostic interpretability (Q2–Q6): a grade of 1 indicates poor image quality and that the image is not usable, 2 indicates restricted image quality and clear loss of information, 3 indicates sufficient image quality, 4 represents good image quality, while 5 represents excellent image quality.^34^

The occurrence of score 3 to 5 for each question (general state or area) and each case (set of imaging parameters) was tallied. Multinominal regressions were performed (package nnet, version 4.2.2, 2022‐10‐31, The R foundation for Statistical Computing) for each of the six questions using kVp, keV, IQ, iMAR, kernel, and slice thickness as predictors. iMAR, keV, and kernel were treated as non‐ordered factors, with iMAR = none, keV = 40, and kernel Qr36f as reference levels. The answers to the questions (score 1−5) were treated as ordered data. For each predictor, a *p*‐value was calculated where a *p*‐value < 0.05, was set to indicate statistical significance, indicating that the parameter has an impact on the image quality. The regression coefficient *β*≠0, and the log odd, the ratio of probability for a score to the probability of score 1 can be calculated using the Equation (1).

(1) $$\begin{array}{ccc} & & \ln\left(\frac{P\left(score=2,3,4\;or\;5\right)}{P\left(score=1\right)}\right)=\beta_{0}+\beta_{1}kVp\\ & & +\sum_{keV_{i}}^{keV_{n}}\beta_{2i}keV_{i}+\beta_{3}IQ+\sum_{IMAR_{i}}^{IMAR_{n}}\beta_{4i}IMAR_{i}\\ & & +\sum_{kernel_{i}}^{kernel_{n}}\beta_{5i}kernel_{i}+\beta_{6}slice\;thickenss \end{array}$$

Inter‐reader agreement was calculated using IntraClass Correlation (ICC) function, two‐way random effects model, average consistency from the irr package in R. Then inter‐reader agreement can be given as Equation (2)

(2) $$ICC{\left(C,k\right)}_{inter}=\frac{MS_{R}-MS_{E}}{MS_{R}}$$

where MS_R_ is the mean square for between subjects, MS_E_ is the mean square error. Similarly, the intra‐reader agreement was assessed by calculating ICC using average agreement.^35^ ICC for intra reader agreements can be given as Equation (3)

(3) $$ICC{\left(2,k\right)}_{intra}=\frac{MS_{R}-MS_{E}}{MS_{R}+\frac{MS_{C}+MS_{E}}{n}}$$

where MS_R_, MS_E_ is as for Equation (2) and MS_C_ is means square between measurements (raters), and *n* is the number of cases/subjects.^36^

#### 2.3.2 Three methods to quantify metal artifacts

Three distinct approaches were used for metal artifact quantification: manual ROI analysis, Fourier method for streak evaluation, and blooming artifact quantification by segmenting the titanium in the LVAD. These three approaches resulted in four parameters, Diff_HU_, SD_ARTIFACT_, AmplitudeLowFreq, and BloomVol further described below.

##### Artifact ROI analysis

The severity of metal artifacts was objectively quantified by computing the absolute deviation in mean attenuation, measured in HU, between an ROI located where the artifact was most pronounced and a comparable structure without the artifact (Diff_HU_). A larger absolute HU deviation indicates a more pronounced metal artifact.^17^, ^37^ The ROI was consistently placed in the same region and slice location across all CT image cases. Furthermore, the standard deviation (SD) of the ROI, situated where the metal artifact was most pronounced (SD_ARTIFACT_), could potentially serve as an indicator of artifact extent. Here, higher SD values signify a more substantial artifact load.^38^

Linear regression models were used to analyze the relationship between the quantity and the predictors such as kVp, keV, kernel, iMAR, slice thickness, CTDIvol (computed tomography dose index volume), and IQ. The model that provided the best description of the quantity was determined using R and the built‐in step AIC (Akaike Information Criteria) function, as well as an analysis of variance (ANOVA) table. The coefficients for each level of the predictor (factor), except the reference level, represent the percentage change in the predicted value of the quantity when the factor level changes, while keeping all other variables constant. To determine the equation for an optimized model describing the Diff_HU_, we employed a linear fit for the phantom and a logarithmic linear fit for the patient. Similarly, the model for SD_ARTIFACT_ was based on a logarithmic linear fit for both the phantom and the patient.

##### Quantifying streak artifacts by the Fourier method

The Fourier method derives from the observation that metal objects cause alternating patterns of bright and dark streaks, that can be quantified by a method involving measuring CT values along a contour surrounding the metal, as previously described by Mangold and Hokamp.^35^, ^39^, ^40^ The CT values then undergo a frequency analysis through the discrete Fourier transform. A spectrum with a higher proportion of low frequencies signifies a more intense metal artifact. An in‐house developed Python program was utilized for the analysis, enabling user image selection and automatic contour delineation of the LVAD through thresholding and the “find contour” algorithm. Calculations encompassed three sequential images, with results being averaged. We plotted the HU along the contour, the Fourier of HU along the contour and the corresponding. The amplitude of low frequency, bin1‐2 (AmplitudeLowFreq) was used for further statistical analysis, such as linear regression (as described in the section ROI analysis), and the associated coefficients of variation (CV) were recorded.

##### Quantifying blooming artifacts by the Volume method

Blooming artifacts arise when metal objects, within imaging, seem larger than their actual size. Therefore, the spatial magnitude of the blooming can be deduced from the depicted object's size,^40^, ^41^, ^42^, ^43^ and in this study, it was quantified by determining the LVAD's volume (BloomVol) displayed in the CT images. ParaView 5.11.0 facilitated volume measurement through segmentation. The process incorporated three filtrations:

1. Initial threshold filtration set a lower boundary at 800 for the phantom (and 2500 for the patient) and an upper boundary of 32 762.
2. Connectivity filtration was employed in “extract largest region” mode.
3. The “integrate variable” function computed the volume.

**FIGURE 4:**
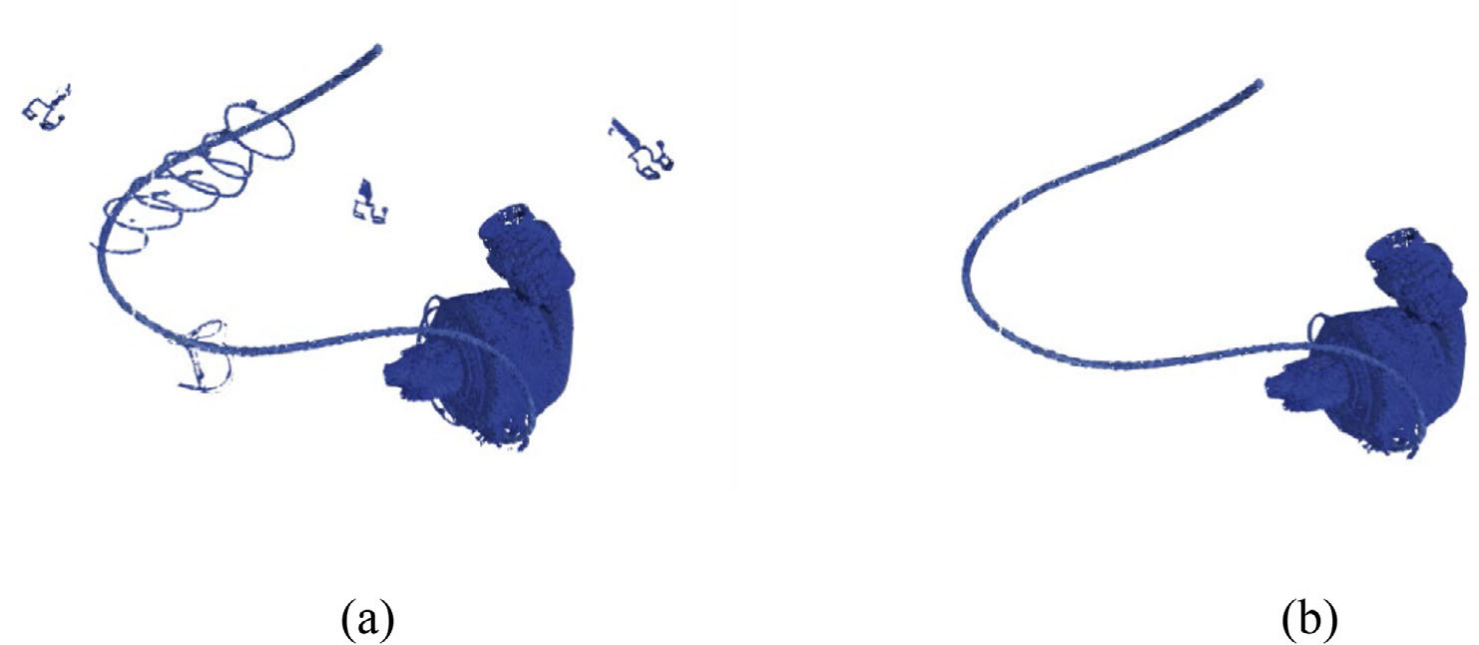
(a) Segmentation after the thresholding of a patient image with kernel Qr56, and (b) after application of connectivity filtration. The BloomVol was obtained by the integrate variable.

Figure 4 offers a visual representation of the segmentation process.

Log‐linear regression was employed, as described in the section for ROI analysis, to determine the model for BloomVol for both phantom and patient data set.

## 3 RESULTS

### 3.1 Qualitative evaluation: Absolute visual grading

The highest overall diagnostic score, obtained by summing the grades 3 to 5 for all six questions (VG_SUM_), was 25 for the phantom image cases acquired with both 120 and 140 kVp, IQ 80 and reconstructed with 3 mm slice thickness, quantitative regular kernel, level 40 (Qr40f), iMAR with pacemaker preset and 110 keV (Cases 34 and 36, Table 2). For thin slices (0.4 mm), the achieved highest score was 22, which was obtained for images acquired at 140 kVp, and the same IQ, reconstructing kernel, iMAR preset and keV (Case 8). When examining metal artifacts in general (Q1) separately, the visual grading showed the same results as for Q2–Q6, except that body‐vascular kernel level 40 (Bv40f) also exhibited the fewest metal artifacts (Case 1). For diagnostic interpretability, summing the grades 3 to 5 for Q2–Q6 the poly‐energetic reconstruction (T3D, 20−120 keV), Qr40f, 0.4 mm slice thickness (Case 28), and 110 keV, Qr40f and 3 mm thickness are preferred for the phantom (Cases 34 and 36, Table 2).

**TABLE 2:** Description of the phantom image cases and results from the assessment of metal artifacts.

	kVp	IQ	Kernel	iMAR	VMI (keV)	Slice Thickness (mm)	Diff_HU_ (metal‐normal)	SD_ARTIFACT_ (HU)	BloomVol (cm^3^)	AmplitudeLowFreq	CV (%)	Q1	Q2	Q3	Q4	Q5	Q6	VG_SUM_
Case 1	120	80	Bv40f	Ex	110	0.4	−86	50	61.6	42723.6	5.4	5	1	1	2	3	3	15
Case 2	120	80	Bv40f	Hi	110	0.4	−66	67	61.2	43578.6	2.3	4	1	3	2	3	4	17
Case 3	120	80	Bv40f	Th	110	0.4	−131	141	61.7	44911.3	3.9	2	1	2	0	0	2	7
Case 4	120	80	Bv40f	None	110	0.4	−213	128	62.6	40267.9	13.8	1	4	0	0	3	4	12
Case 5	120	80	Bv40f	Pa	110	0.4	−134	107	61.6	38909.2	6.6	3	2	3	2	3	4	17
Case 6	120	100	Qr40f	Pa	110	0.4	−119	151	59.9	44501.0	11.0	3	2	3	2	4	3	17
Case 7	140	55	Qr40f	Pa	110	0.4	−99	99	59.1	52724.3	9.5	4	5	3	1	4	1	18
Case 8	140	60	Qr40f	Pa	110	0.4	−51	140	58.5	38961.9	3.4	5	4	3	1	4	5	22
Case 9	120	55	Qr40f	Pa	110	0.4	−137	168	61.3	52770.9	4.0	3	2	3	1	4	5	18
Case 10	120	60	Qr40f	Pa	110	0.4	−108	150	60.0	39782.8	5.3	3	2	3	2	4	4	18
Case 11	120	80	Bl56f	Pa	110	0.4	−45	145	42.3	52021.7	3.5	4	3	2	1	2	1	13
Case 12	120	80	Bv36f	Pa	110	0.4	−115	133	62.7	46673.0	13.0	4	3	3	1	3	4	18
Case 13	120	80	Bv40f	Pa	110	0.4	−72	120	61.8	41604.8	4.3	2	3	3	2	3	4	17
Case 14	120	80	Bv44f	Pa	110	0.4	−103	108	61.2	42936.0	1.6	4	2	3	1	3	4	17
Case 15	120	80	Bv56f	Pa	110	0.4	−99	138	56.0	44470.7	5.7	3	1	1	0	2	2	9
Case 16	120	80	Qr36f	Pa	110	0.4	−133	123	60.6	42913.5	2.2	4	4	3	1	3	3	18
Case 17	120	80	Qr40f	Pa	110	0.4	−115	118	60.2	43677.7	1.8	2	4	4	3	4	3	20
Case 18	120	80	Qr44f	Pa	110	0.4	−132	126	58.5	43448.0	3.8	3	4	3	1	4	3	18
Case 19	120	80	Qr40f	Pa	40	0.4	−109	207	89.8	37437.5	5.4	0	2	1	0	1	0	4
Case 20	120	80	Qr40f	Pa	62	0.4	−86	134	84.6	39828.3	5.7	0	4	1	0	3	3	11
Case 21	120	80	Qr40f	Pa	190	0.4	−108	158	52.6	47422.1	1.1	2	2	3	1	2	3	13
Case 22	120	80	Bv40f	None	70	1	−150	165	80.0	38917.6	0.8	2	4	3	2	5	5	21
Case 23	120	80	Qr40f	None	70	1	−143	170	NA	40270.2	1.3	3	5	1	2	3	3	17
Case 24	120	80	Qr40f	None	T3D	0.4	46	211	83.3	43657.8	3.0	0	1	1	1	4	3	10
Case 25	140	80	Qr40f	Pa	40	0.4	−273	275	92.7	45531.2	7.0	0	3	0	0	1	1	5
Case 26	140	80	Qr40f	Pa	67	0.4	−141	143	81.5	46290.3	7.0	1	3	0	0	1	1	6
Case 27	140	80	Qr40f	Pa	110	0.4	−121	96	59.6	56096.3	5.5	3	4	3	1	3	3	17
Case 28	140	80	Qr40f	Pa	T3D	0.4	−142	129	75.0	55114.1	3.4	3	6	3	3	5	3	23
Case 29	90	80	Qr40f	Pa	110	0.4	−14	147	65.2	45112.8	2.2	1	0	3	0	2	1	7
Case 30	90	80	Qr40f	Pa	T3D	0.4	−170	199	85.0	45160.2	7.1	0	4	1	0	2	1	8
Case 31	120	80	Bv40f	Pa	T3D	0.4	−140	141	79.0	49692.4	3.0	3	5	3	1	4	3	19
Case 32	120	80	Qr40f	Pa	110	0.4	−39	137	60.3	48914.2	1.5	3	4	3	1	4	4	19
Case 33	120	80	Qr40f	Pa	110	1	−123	108	60.3	50246.3	2.4	3	5	3	3	3	3	20
Case 34	120	80	Qr40f	Pa	110	3	−136	108	61.8	48888.0	3.7	5	4	6	2	5	3	25
Case 35	140	80	Qr40f	Pa	110	1	−97	89	59.4	51022.6	2.7	4	5	3	3	4	3	22
Case 36	140	80	Qr40f	Pa	110	3	−140	81	60.8	51012.5	5.4	5	5	3	3	5	4	25

*Note*: The acquisition parameters, kVp and IQ (image quality) level, and the reconstruction parameters, kernel, iMAR (iterative metal artifact reduction), and virtual mono‐energetic image (VMI), varied across the image cases. The results from manual ROI analyses included the difference in Hounsfield Units (HU) between areas with metal artifacts and normal tissue (Diff_HU_—Metal minus normal), and the standard deviation in areas affected by metal artifacts (SD_ARTIFACT_). The volume from the blooming assessment (BloomVol) and the results from the streak artifact assessment, the amplitude of low frequencies in bin 1−2 (AmplitudeLowFreq) and the associated coefficient of variation (CV). The occurrence of grade 3, 4, and 5 responses for visual grading of metal artifact (Q1) and diagnostic interpretability (Q2–Q6), and their respective sums (VG_SUM_).

In the multinominal regression analysis of the visual grading results, considering the acquisition and reconstructions parameters for the phantom images, it was observed that selection of the virtual mono‐energetic (keV) had a more pronounced impact on the grading of Q1–Q6 than the selection of kVp. This was indicated by a higher relative probability ratio given by Equation (1). Selecting 110 keV seems to elevate the probability of achieving a score of 4 for all questions, except Q5 (spinal area). The choice of iMAR preset for extremities and hip implants is most effective to reduce metal artifacts (Q1). However, these iMAR presets result in a reduction of the image quality as reflected in Q4–Q6 (lung tissue and spinal cord). Kernel Qr44f and Bv56f are most effective in mitigating metal artifacts (Q1). The selection of kernel appears to have negligible impact when assessing the lumen of the LVAD (Q2). For the evaluation of cardiac tissue (Q3), Bv36f proves to be the optimal choice, Qr40f for lung tissue (Q4–Q5) and spinal area (Q6). Increasing the slice thickness has a positive effect on the assessment, particularly for Q2 and Q5 (see Table A1).

In the in vivo dataset, the highest score achieved was 14, resulting from the reconstruction of 0.8 and 3 mm slice thickness and pacemaker preset, kernel Bv56f, and T3D (Cases 26 and 27, Table 3). For 0.4 mm slices, a score of 11 was achieved for two settings, kernel Qr40f, 110 keV along with iMAR preset for thoracic coils and hip implants (Cases 18 and 20). For addressing metal artifacts (Q1), 110 keV, body‐lung level 56 (Bl56f), 0.4 mm slice thickness, and T3D, Bv56f, 0.8/3 mm slice thickness are preferable (Cases 12, 26, and 27). However, for diagnostic interpretability Q2–Q6, the Bv56f and the same setting as for Q1 proved to be preferable (Cases 26 and 27).

**TABLE 3:** Description of the patient image cases and results from the assessment of metal artifacts.

	Kernel	iMAR	VMI (keV)	Slice thickness (mm)	Diff_HU_ (metal‐normal)	SD_ARTIFACT_ (HU)	BloomVol (cm^3^)	AmplitudeLowFreq	CV (%)	Q1	Q2	Q3	Q4	Q5	Q6	VG_SUM_
Case 1	Qr40f	Pa	40	0.4	776	187	96.3	69478.9	12.4	0	0	0	0	1	0	1
Case 2	Qr40f	Pa	62	0.4	396	121	73.5	37266.1	1.5	0	0	0	0	0	0	0
Case 3	Qr40f	Pa	T3D	0.4	331	75	54.7	23155.9	1.1	1	0	1	0	3	4	9
Case 4	Qr40f	Pa	SSP‐70	0.4	446	113	62.5	40961.9	11.4	0	0	0	0	3	0	3
Case 5	Qr40f	Pa	90	0.4	308	59	49.1	26209.7	2.6	0	0	0	0	2	1	3
Case 6	Bv56f	Pa	90	0.4	428	104	46.4	45695.2	9.4	0	0	0	0	3	1	4
Case 7	Qr40f	Pa	190	0.4	304	80	35.3	35855.6	5.2	0	0	0	0	2	2	4
Case 8	Qr36f	Pa	110	0.4	339	137	43.2	21071.3	19.1	1	0	0	0	4	2	7
Case 9	Qr40f	Pa	110	0.4	268	77	42.8	18676.3	14.1	0	0	0	0	3	1	4
Case 10	Qr44f	Pa	110	0.4	360	75	42.4	31958.9	14.1	1	0	0	0	3	1	5
Case 11	Qr56f	Pa	110	0.4	397	104	40.2	40797.9	11.7	0	0	0	0	2	2	4
Case 12	Bl56f	Pa	110	0.4	355	102	37.2	51823.4	13.8	2	0	0	0	3	2	7
Case 13	Bv36f	Pa	110	0.4	350	134	43.5	22780.7	10.3	0	0	0	0	2	1	3
Case 14	Bv40f	Pa	110	0.4	286	58	43.1	21459.0	16.8	0	0	0	0	4	2	6
Case 15	Bv44f	Pa	110	0.4	340	70	42.8	39596.0	9.8	0	0	0	0	2	2	4
Case 16	Bv56f	Pa	110	0.4	373	88	40.6	44783.1	14.0	0	0	0	0	0	0	0
Case 17	Qr40f	None	110	0.4	86	37	42.9	37359.9	4.9	0	0	0	0	3	0	3
Case 18	Qr40f	Tc	110	0.4	211	94	42.8	15596.6	22.8	1	1	1	2	5	1	11
Case 19	Qr40f	Ei	110	0.4	416	106	42.8	17471.6	13.3	1	0	1	1	4	3	10
Case 20	Qr40f	Hi	110	0.4	398	108	42.8	16930.1	17.9	1	0	1	1	4	4	11
Case 21	Bv56f	Tc	110	0.4	127	64	40.6	57752.4	12.3	0	0	0	0	4	1	5
Case 22	Bv56f	Pa	110	1	411	98	34.4	39611.4	12.3	0	0	1	1	5	3	10
Case 23	Qr40f	Pa	110	1	277	77	36.3	17451.3	3.2	1	0	0	0	4	2	7
Case 24	Bv56f	Pa	110	3	317	89	27.0	29432.3	32.6	1	0	1	2	4	3	11
Case 25	Qr40f	Pa	110	3	264	75	28.7	15987.0	26.2	0	0	0	1	5	1	7
Case 26	Bv56f	Pa	T3D	0.8	321	101	50.1	35135.6	5.0	2	2	2	1	5	2	14
Case 27	Bv56f	Pa	T3D	3	342	102	37.1	28447.4	19.9	2	2	2	1	4	3	14

*Note*: The acquisition parameters were IQ (image quality) level 80 and 120 kVp. The reconstruction parameters, kernel, iMAR (iterative metal artifact reduction), virtual mono‐energetic image (VMI) and slice thickness, varied across the image cases. The results from manual ROI analyses included the difference in Hounsfield Units (HU) between areas with metal artifacts and normal tissue (Diff_HU_—Metal minus normal), and the standard deviation in areas affected by metal artifacts (SD_ARTIFACT_). The volume from the blooming assessment (BloomVol) and the results from the streak artifact assessment, the amplitude of low frequencies in bin 1−2 (AmplitudeLowFreq) and the associated coefficient of variation (CV). The occurrence of grade 3, 4 and 5 responses for visual grading of metal artifact (Q1) and diagnostic interpretability (Q2–Q6), and their respective sums (VG_SUM_).

In the multinominal regression analysis of patient image visual grading, it was observed that 110 keV and T3D had a significantly positive effect on both metal artifacts (Q1) and on diagnostic interpretability, Q2–Q6. The iMAR preset for Extremities seems to ameliorate metal artifacts (Q1), but shows contrasting effect on Q2, Q4, and Q6. Specifically, for the evaluation of lumen of LVAD (Q2) pacemaker and thoracal presets are preferable. For the lung opposite to the LVAD (Q5), it appeared that either the extremities or hip implants presets offered the best image quality. The kernels Bl56f and Bv44f reduce the metal artifacts (Q1). However, for the lumen (Q2) the kernels did not significantly impact grading and were therefore excluded from the model. For cardiac tissue (Q3), kernel Bv56f and Qr40f have a positive impact. The kernels had a statistically significant impact on the Q5 (right lung), with Bv40f likely providing the best image quality, while Bv56f improved the image quality in the spine area (Table A2).

The inter‐reader reliability, expressed as ICC (intraclass correlation coefficient), ranged from 0.54 to 0.85 for the different questions in the phantom study, and similar range of 0.24 to 0.7 for the patient. See Table 4 for ICC values, *p*‐values and confidence intervals associated with all the questions (Q1–Q6).

**TABLE 4:** Inter reader agreement for phantom study and the patient calculated by ICC two way consistency average.

	Q1	Q2	Q3	Q4	Q5	Q6
**Phantom**						
**ICC**	0.852	0.538	0.782	0.653	0.696	0.631
***p*‐value**	< 0.001	< 0.001	< 0.001	< 0.001	< 0.001	< 0.001
**CI**	(0.722, 0.911)	(0.285, 0.723)	(0.664, 0.87)	(0.463, 0.792)	(0.53, 0.818)	(0.43, 0.779)
**Patient**						
**ICC**	0.539	0.239	0.363	0.524	0.698	0.405
***p*‐value**	0.0024	0.161	0.0526	0.00347	< 0.001	
**CI**	(0.205, 0.763)	(−0.312, 0.61)	(−0.098, 0.673)	(0.18, 0.756)	(0.479, 0.845)	(−0.025, 0.695)

Abbreviations: CI, confidence interval; ICC, IntraClass Correlation.

In terms of intra‐reader reliability, all questions except Q2 had a higher ICC value than 0.86. The highest intra‐reader reliability was observed for Q1 with an ICC of 0.981, *p*‐value of 4.37e‐10, and a confidence interval (CI) of (0.935, 0.997). On the other hand, the lowest intra‐reader reliability was found for Q2 with ICC = 0.678, *p* = 0.0427 and CI = (−0.181, 0.951).

### 3.2 Metal artifact quantification

#### 3.2.1 ROI analyses

In the phantom cases, we found that the HU value of lung tissue affected by metal artifacts was lower than that of normal tissue, while the opposite was observed in‐vivo. Among the iMAR presets, kernels, IQ settings, and keV levels, the minimum Diff_HU_ was observed for 90 kVp, the pacemaker preset, the Qr40f kernel, IQ80, and 110 keV in the phantom datasets. In the in‐vivo data, the minimum Diff_HU_ was found for 120 kVp, no IMAR, the Qr40f kernel, and 110 keV. Similarly, the SD_ARTIFACT_ was minimized in the phantom cases with 120 kVp, the extremities iMAR, the Bv40f kernel, IQ80, and 110 keV. In the in‐vivo datasets, the lowest SD_ARTIFACT_ was found in the same image settings as for Diff_HU_ (refer to Table 2 and Table 3).

**FIGURE 5:**
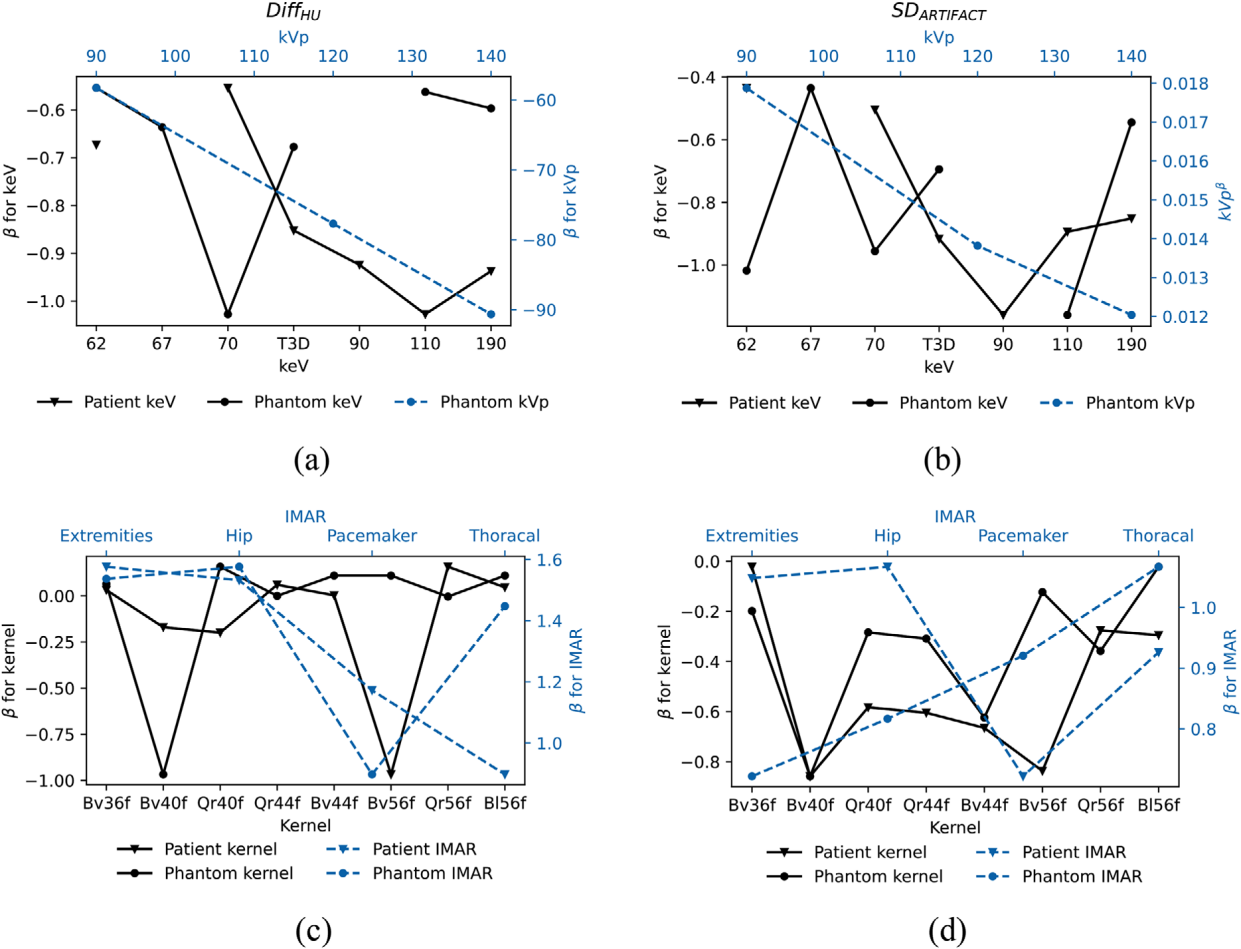
The change (*β*) in (a) Diff_HU_ and in (b) SD_ARTIFACT_ as function of keV relative to 40 keV and kVp. The change (*β*) in (c) Diff_HU_ and in (d) SD_ARTIFACT_ as function of kernel relative to Qr36f and as function of iMAR relative to no iMAR.

Both Diff_HU_ and SD_ARTIFACT_ are influenced by the selection of iMAR presets and kernels (see Figure 5c–d). In the in‐vivo dataset, the thoracal preset reduced Diff_HU_ the most, while the pacemaker preset had the most pronounced reduction for the phantom. However, the pacemaker preset reduced SD_ARTIFACT_ mostly for the patient, and the extremity preset for the phantom. Increasing kVp decreased both Diff_HU_ and SD_ARTIFACT_. This trend was similar for keV, although there were some outliers as indicated in Figure 5a–b. However, the predictors were not as effective in describing Diff_HU_ as they were for SD_ARTIFACT_ (refer to adjusted *R*‐squared in Table 5) and the model adequacy is better for the patient data. Increasing slice thicknesses and dose (IQ) reduced the SD_ARTIFACT_ as expected, refer to Table A3 for all regression coefficients and associated *p*‐values for the models given in Table 5.

**TABLE 5:** Regression model for two approaches to measure metal artifacts: DiffHU and SDARTIFACT, both based on ROI analyses.

	Phantom		Patient	
Measured quantity	Optimized regression model	Model adequacy (Adjusted‐*R^2^*)	Optimized regression model	Model adequacy (Adjusted‐*R^2^*)
**Diff_HU_** Deviation between HU in lung tissue with and without metal artifact	$Diff_{HU}=\beta_{0}+\beta_{1}kVp+\beta_{2}keV+\beta_{3}sw+\beta_{4}IMAR+\beta_{5}kernel+\beta_{6}IMAR:kernel+\beta_{7}kernel:keV+\varepsilon$	0.280	$Diff_{HU}=keV^{\beta 1}\cdot sw^{\beta 2}\cdot IMAR^{\beta 3}\cdot kernel^{\beta 4}\cdot IMAR:kernel^{\beta 5}\cdot kernel:keV^{\beta 6}\cdot e^{\left(\beta 0+\varepsilon \right)}$	0.949
**SD_ARTIFACT_** Standard deviation in lung tissue with metal artifact.	$SD_{ARTIFACT}=kVp^{\beta 1}\cdot keV^{\beta 2}\cdot IQ^{\beta 3}\cdot sw^{\beta 4}\cdot IMAR^{\beta 5}\cdot kernel^{\beta 6}\cdot IMAR:kernel^{\beta 7}\cdot kernel:keV^{\beta 8}\cdot e^{\left(\beta 0+\varepsilon \right)}$	0.755	$SD_{ARTIFACT}=keV^{\beta 1}\cdot sw^{\beta 2}\cdot IMAR^{\beta 3}\cdot kernel^{\beta 4}\cdot IMAR:kernel^{\beta 5}\cdot kernel:keV^{\beta 6}\cdot e^{\left(\beta 0+\varepsilon \right)}$	0.982

#### 3.2.2 Streak artifacts

**FIGURE 6:**
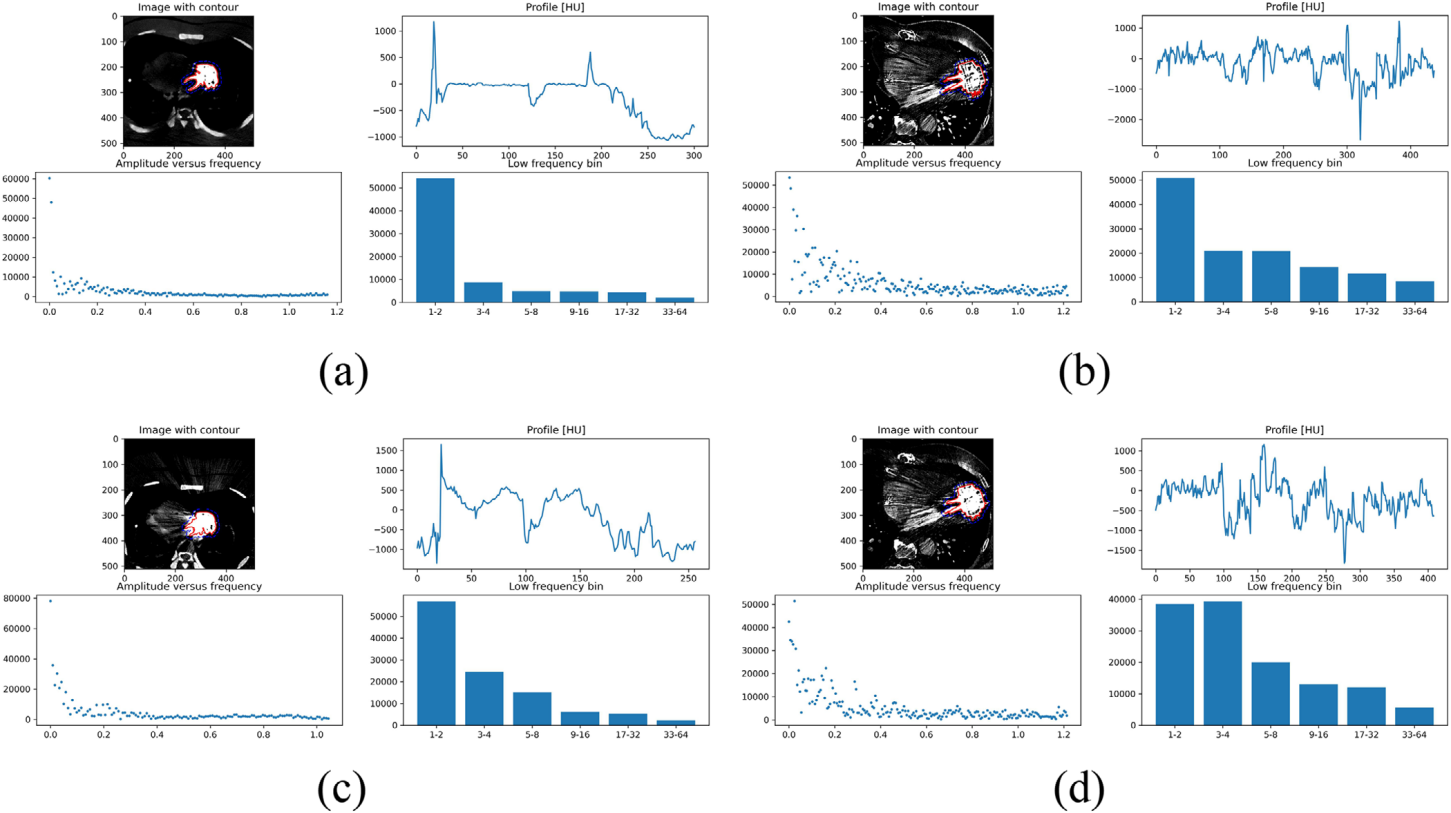
An exemplification of the Fourier representation exhibiting high image quality, as per visual grading results: (a) Case 36 for phantom and (b) Case 12 for the patient. Conversely, examples of suboptimal image quality (c) phantom, Case 24 and (d) for the patient, Case 16.

The amplitude for the sum of bin 1−2 (AmplitudeLowFreq), which corresponds to frequencies ranging from 0 to 0.0142, is presented in Table 2 and Table 3, along with the associated coefficient of variation. Figure 6 displays the contour and frequency plot for both phantom and patient images, categorized as good and bad based on visual grading analyses. The lowest AmplitudeLowFreq is observed for 120 kVp, pacemaker iMAR, Qr40f kernel, IQ80, and 40 keV in the phantom. In the patient settings, the lowest AmplitudeLowFreq was observed for the iMAR preset for thoracic coils, Qr40f kernel, and 110 keV.

**FIGURE 7:**
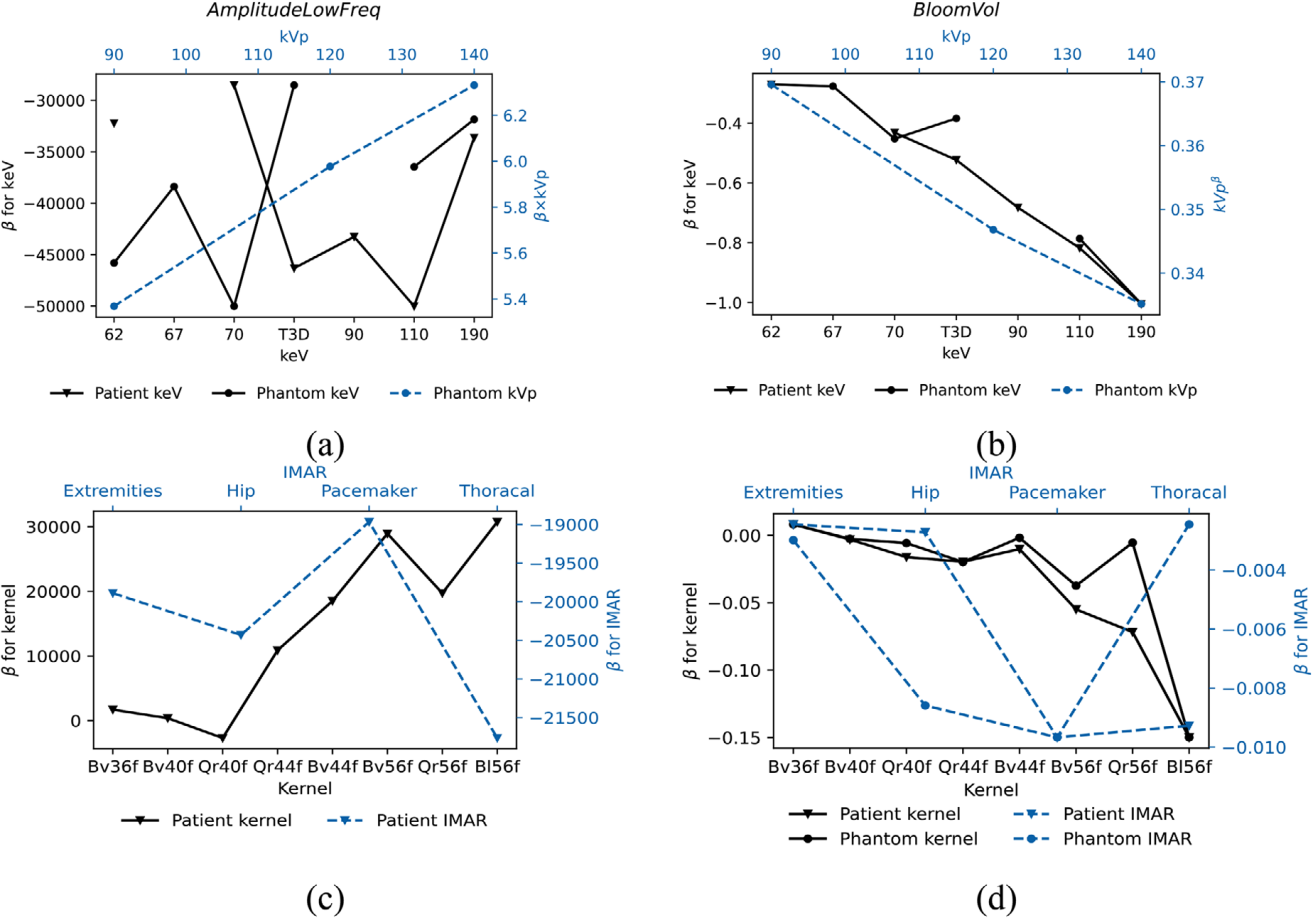
The change (*β*) in (a) AmplitudeLowFreq and in (b) BloomVol as function of keV relative to 40 keV and kVp. The change (*β*) in (c) AmplitudeLowFreq and in (d) BloomVol as function kernel relative to Qr36f and as function of iMAR relative to no iMAR.

Table 6 provides the regression model for the AmplitudeLowFreq. The model adequacy is poor for the phantom data, and excellent for the patient data. In the case of the patient, the AmplitudeLowFreq tends to increase for increasing kVp and decreasing with keV. Sharper kernel tends to increase the AmplitudeLowFreq (refer to Figure 7). See Table A4 for all regression coefficients and associated *p*‐values for the models given in Table 6.

**TABLE 6:** Regression model for assessing metal streak artifacts by AmplitudeLowFreq, and blooming artifact by BloomVol.

	Phantom		Patient	
Measured quantity	Optimized regression model	Model adequacy (Adjusted‐*R* ^2^)	Optimized regression model	Model adequacy (Adjusted‐*R^2^*)
**AmplitudeLowFreq** Streak artifacts by amplitude of low frequencies bin 1−2	$AmplitudeLowFreq=\beta_{0}+\beta_{1}kVp+\beta_{2}keV+\beta_{3}sw+\varepsilon$	0.2937	$AmplitudeLowFreq=\beta_{0}+\beta_{1}keV+\beta_{2}sw+\beta_{3}kernel+\beta_{4}IMAR+\beta_{5}keV:sw+\beta_{6}keV:kernel+\varepsilon$	0.9952
**BloomVol** Spatial distortion by volumetric representation of LVAD	$BloomVol=kVp^{\beta 1}\cdot keV^{\beta 2}\cdot kernel^{\beta 3}\cdot imar^{\beta 4}\cdot slice^{\beta 5}\cdot e^{\left(\beta 0+\varepsilon \right)}$	0.9893	$BloomVol=\cdot keV^{\beta 1}\cdot kernel^{\beta 2}\cdot imar^{\beta 3}\cdot slice^{\beta 4}\cdot e^{\left(\beta 0+\varepsilon \right)}$	0.9889

#### 3.2.3 Blooming artifacts

The LVAD volume (BloomVol) ranged from a minimum of 42.3 to 92.7 cm^3^ in the phantom datasets, as presented in Table 2. In the in‐vivo datasets, the BloomVol varied from 27.0 to 96.3 cm^3^ as presented in Table 3. The optimized model for BloomVol with respect to kVp, keV, kernel, and iMAR is given by the equations in Table 6. The BloomVol can be adequately described by the parameters included in the model, with an adjusted *R^2^* value of 0.9893 for the phantom and 0.9889 for the patient.

Sharper kernels with higher kernel numbers contribute to a smaller BloomVol, as do higher kVp and keV values. The selection of iMAR does not have a statistically significant effect on BloomVol (except for the pacemaker iMAR on phantom images), nor do CTDIvol and IQ. Therefore, the blooming artifact is dependent on the following factors, ranked from the largest impact to the least impact: kVp, kernel, keV, and iMAR. The change in BloomVol as a function of keV, kV, iMAR, and kernel is illustrated in Figure 7. The impact of slice thickness was not significant in the model.

### 3.3 Correlation between different assessments methods

In the phantom datasets, there was a negative correlation of −0.39 between the VG_SUM_ and the SD_ARTIFACT_. Additionally, there was a correlation between the VG_SUM_ and the BloomVol, with a correlation coefficient of −0.31. However, the correlation between Q1 (metal artifacts) and SD_ARTIFACT_ was stronger, with correlation coefficients of −0.58, and −0.53 between Q1 and BloomVol. Furthermore, there was a moderate negative correlation between Q3 (cardiac tissue) and SD_ARTIFACT_ (−0.33), as well as the BloomVol (−0.34).

In the in‐vivo datasets, there was a negative correlation between the VG_SUM_ and the BloomVol, with a correlation coefficient of −0.40. There was also a correlation of −0.48 between the VG_SUM_ and the AmplitudeLowFreq, a correlation between the AmplitudeLowFreq and the assessment of the lung closest to the LVAD (Q4), with a correlation coefficient of −0.43. Additionally, there was a correlation between the AmplitudeLowFreq and the assessment of lung tissue (Q5), with a correlation coefficient of −0.49. The correlation between Diff_HU_ and the visual grading was weak for all questions for both the phantom and patient (refer to Table 7).

**TABLE 7:** For phantom and patient study: Correlation between visual grading (score for Q1–Q6, and the sum of score VG_SUM_) and objective metal artifact assessment DiffHU (HU), SD_ARTIFACT_ (HU), BloomVol (cm^3^) and AmplitudeLowFreq.

Phantom	VG_SUM_		Q1		Q2		Q3		Q4		Q5		Q6		Diff_HU_		SD_ARTIFACT_		BloomVol		Amplitude LowFreq	
Patient	*rho*	*p*‐value	*rho*	*p*‐value	*rho*	*p*‐value	*rho*	*p*‐value	*rho*	*p*‐value	*rho*	*p*‐value	*rho*	*p*‐value	*rho*	*p*‐value	*rho*	*p*‐value	*rho*	p‐value	*rho*	*p*‐value
**VG_SUM_**			0.626	**0.000**	0.607	**0.000**	0.790	**0.000**	0.742	**0.000**	0.816	**0.000**	0.569	**0.000**	−0.084	0.594	−0.389	**0.010**	−0.305	**0.050**	0.105	0.503
**Q1**	0.772	**0.000**			0.198	0.204	0.480	**0.001**	0.489	**0.001**	0.410	**0.006**	0.363	**0.017**	0.163	0.295	−0.576	**0.000**	−0.529	**0.000**	0.126	0.422
**Q2**	0.535	**0.004**	0.534	**0.004**			0.230	0.139	0.285	0.064	0.418	**0.005**	0.110	0.482	−0.411	**0.006**	−0.261	0.091	−0.083	0.600	0.288	0.061
**Q3**	0.807	**0.000**	0.639	**0.000**	0.638	**0.000**			0.619	**0.000**	0.603	**0.000**	0.437	**0.003**	0.144	0.358	−0.333	**0.029**	−0.344	**0.026**	0.108	0.489
**Q4**	0.767	**0.000**	0.470	**0.013**	0.551	**0.003**	0.821	**0.000**			0.646	**0.000**	0.425	**0.005**	0.086	0.582	−0.366	**0.016**	−0.243	0.121	−0.056	0.723
**Q5**	0.849	**0.000**	0.489	**0.010**	0.439	**0.022**	0.587	**0.001**	0.719	**0.000**			0.479	0.001	−0.036	0.819	−0.204	0.190	−0.137	0.385	0.026	0.868
**Q6**	0.785	**0.000**	0.571	**0.002**	0.145	0.470	0.651	**0.000**	0.458	**0.016**	0.471	**0.013**			−0.002	0.992	−0.265	0.086	−0.146	0.355	−0.254	0.100
**Diff_HU_**	−0.159	0.427	−0.069	0.731	−0.201	0.315	0.124	0.537	−0.090	0.655	−0.323	0.101	0.103	0.609			−0.110	0.483	−0.350	**0.023**	−0.112	0.476
**SD_ARTIFACT_**	−0.011	0.955	0.169	0.401	0.086	0.669	0.136	0.499	0.135	0.501	−0.111	0.583	−0.002	0.991	0.457	**0.016**			0.370	**0.016**	−0.260	0.092
**BloomVol**	−0.396	**0.0410**	−0.149	0.459	0.044	0.829	−0.111	0.580	−0.358	0.067	−0.340	0.083	−0.410	**0.034**	0.398	**0.040**	0.277	0.162			−0.224	0.154
**AmplitudeLowFreq**	−0.477	**0.0120**	−0.338	0.0843	−0.198	0.323	−0.318	0.107	−0.433	**0.024**	−0.487	**0.010**	−0.350	0.074	0.293	0.138	0.100	0.621	0.098	0.628		

Abbreviations: SD_ARTIFACT_ **_,_** standard deviation in areas affected by metal artifacts; VG_SUM_, visual grading of metal artifact.

### 3.4 Examples of patient image cases

**FIGURE 8:**
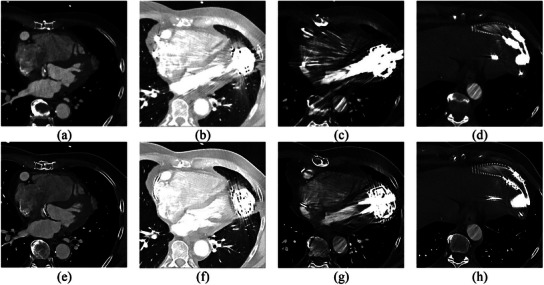
An example of the lowest image quality, Case 2 (62 keV, Qr40f, 0.4 mm) and highest image quality Case 27 (T3D, Bv56f, 3 mm) of: (a) and (e) cardiac tissue, (b) and (f) lung tissue, (c) and (g) inflow cannula and (d) and (h) outflow cannula.

Figure 8 shows the PCCT patient images with highest and lowest total score from visual grading for cardiac and lung tissue, as well as the inflow and outflow cannula. The inflow cannula (Q2) and cardiac tissue (Q3) obtained the highest score with Bv56 and T3D, as for the total score. However, the lung tissue closest to the LVAD was best visualized using 110 keV and kernel Qr40f or Bv56f, while the other lung has high score for both T3D, Bv56 and 110 keV, Qr40f. The spinal cord area achieved the highest score for 110 keV, Bv56, and iMAR with hip preset.

## 4 DISCUSSION

In this study, we optimized PCCT for cardiac imaging in LVAD patients, where the proximity of metal close to the region of interest adversely affects image quality. We evaluated 36 different exposure and reconstruction settings in a thoracic phantom and 27 reconstructions in an in‐vivo dataset of a patient with an LVAD implant. Six radiologists visually assessed the images and metal artifacts were assessed using three established objective quantitative methods.

Regarding the three quantitative metal artifact assessment methods—artifact HU analysis, Fourier analysis, and blooming volume—the model adequacy was moderate for the phantom datasets, except for the blooming artifact quantification, which was well‐fitted. Conversely, the in‐vivo data had good fit for all quantities which means that kVp, VMI (keV) level, kernel, iMAR, and slice thickness effectively explain the variations in Diff_HU_, SD_ARTIFACT_, AmplitudeLowFreq, and BloomVol. The absolute HU difference in the in‐vivo datasets was approximately 340, aligning with a prior study examining metal artifacts in the spine, shoulder, or extremity implants.^17^ In the in‐vivo datasets, the difference in HU decreased as the keV increased, showing reduced measured metal artifact (Diff_HU_) with higher keV settings. This concurs with findings by Bamberg et al.^37^ which demonstrated a decrease in absolute HU values with increasing keV. However, the phantom datasets displayed the opposite trend. This inconsistency, coupled with poor model adequacy may stem from the uneven contrast distribution in the cadaver lamb cardiac phantom. Thus, caution is advised when comparing patient and phantom outcomes. Additionally, there is a fundamental difference between the quantitative methods when it comes to the amount of data involved in the analysis. Diff_HU_ and SD_ARTIFACT_ are based on only one image in the series, while AmplitudeLowFreq is an average of three images, and BloomVol is based on the whole image series. Hence BloomVol has better premises to reflect the artifacts throughout the series.

The correlations between the visual grading and three quantitative methods ranged from weak to good, but some inconsistencies were evident here as well between the in‐vivo datasets and the phantom datasets. Notably, there was better inter‐reader agreement for the phantom‐dataset compared to the in‐vivo dataset. This disparity might stem from clearer phantom reading instructions and the fact that some radiologists perhaps focused on the most problematic image in the series, while others averaged their assessment across all images. The correlation between the visual grading of metal artifacts and Diff_HU_ was weak and lacked statistical significance for both phantom and the in‐vivo dataset, aligning with Hokamp et al.^35^ to use the Fourier method. The Fourier method had strong negative correlation to the visual grading of metal artifact and diagnostic interpretability (VG_SUM_) and visual grading for metal artifacts separately (Q1), for the patient, but weak correlation to data for the phantom. Nonetheless, this study's correlation coefficient was good, whereas Hokamp et al.^35^ observed an excellent correlation. Thus, additional patient data may be required to evaluate the efficacy of the Fourier method.

SD_ARTIFACT_ had a strong negative correlation with the VG_SUM_ and the subjectively assessed metal artifacts in phantom datasets. However, the model adequacy was poor for the phantom. For the patient, SD_ARTIFACT_ had a weak or no correlation to the questions in the visual grading. This is in accordance with the findings by Hokamp et al.^35^ that SD_ARTIFACT_ provided slightly better correlation than Diff_HU_ to visual grading. Finally, BloomVol showed strong negative correlation to the VG_SUM_ for both phantom and in‐vivo data and showed a very good model adequacy for both the phantom and patient. Hence, the volume of the metal object, BloomVol, is considered the most robust and accurate quantitative indicator for the severity of metal artifacts, while the Fourier method for assessing streak artifacts may also be effective.

The quantitative as well as visual assessment of the phantom acquisition revealed that optimal image quality was achieved using acquisitions with 120 kVp and IQ 80. Mergen et al.^44^ and Aquino et al.^20^used 120 kVp, and IQ 68 for thoracoabdominal aorta and IQ 50 for a late enhancement cardiac scan, while Euler at al.^45^ chose 120 kVp and IQ 58 for thoracoabdominal aorta. Rajendan et al.^13^ also chose 120 kVp for a coronary CT angiography. Considering the large amount of metal in LVAD patients, a higher IQ is expected to be favorable in this patient group. In the current study, it was noted that the choice of IQ which affects the mAs (higher IQ, higher mAs) had minimal impact on metal artifacts in the phantom. However, CTDIvol was observed to increase linearly up to an IQ of 100, after which it decreased, while effective mAs increased linearly with IQ level, as expected. CTDIvol was lower for IQ 100 since this scan coincidentally was scanned for 60%−80% of the cardiac cycle instead of 0%−100% as for the other IQ levels.

Visual grading analyses of phantom and patient data showed improved image quality for all anatomic areas evaluated using T3D reconstruction. That is in good agreement to the study of Rajendran et al.^13^ where T3D was selected for abdominopelvic and bone images, while they use VMIs for coronary CT. They claim that using 65 keV or higher level of VMIs reduces the calcium blooming artifact, while our study found that 110 keV reduces the LVAD blooming artifact most.

We found that the image quality was affected more by the reconstructed VMI level (keV) than the chosen kVp. This observation may stem from the exclusion of low kVp settings, because an initial exploration showing low kVp settings caused insufficient image quality. However, current results are in alignment with the phantom study by Skornitzke et al.,^46^ who found that the impact of increasing the tube potential to 140 kVp was low for light metals such as titanium and aluminum.

We found that kernel Qr40 was beneficial for the phantom data set, while kernel Bv56 gave the best image quality for the in‐vivo data. Rajendran et al.^13^ applied kernel Bv48 for VMIs and Qr40 for quantitative images (iodine map and virtual none contrast) for coronary arteries. Mergen et al.^44^ reconstructed VMIs at 65 keV, using QIR 3, kernel Qr40, and 1.5 mm slice thickness. Likewise, Rajendran used Qr40 as basis for quantification of extracellular volume. In a phantom study, Skornitzke et al.^46^ compared two kernels using a ROI analysis based on HU numbers. They observed that using kernel body regular 36 (Br36) led to reduced metal artifacts compared to the Br56. However, no significant differences in shape distortion were observed between Br36 and Br56. In contrast, our study found that the Bv56 kernel performed better in terms of visual grading and blooming artifact reduction for in‐vivo data. However, for the phantom dataset, the Bv36 kernel received higher grades compared to the Bv56 kernel. Using PCCT for evaluation of coronary plaques and stents in a phantom model, performed by Rajagopal et al.,^47^ kernel U70f showed to outperform B46f, and in the study performed by Elias et al.,^48^ Bv72 was superior to Bv64, while there was no significant difference between Bv64 and Bv56. The kernel level 36 is expected to produce smoother images than 56, potentially reducing streak artifacts while enhancing blooming artifacts. Small structures consisting of highly attenuating material are sensitive to blooming artifacts, hence sharp kernels, such as Bv72 are preferred.

The volumetric model used for measuring the blooming artifact revealed that iMAR had less effect on image quality than the kVp, kernel and keV. iMAR offers various presets, but none of them are specifically designed for LVAD metal. Consequently, we applied different presets, in contrast to Anhaus et al.,^49^ who exclusively used MAR presets for dedicated metal, adhering to the preset nomenclature. Among the available presets, we found that the pacemaker preset yielded the most favorable results overall. This is in good agreement with Aissa et al.^25^ who found pacemaker preset preferable for LVAD imaging using an energy integrated detector. While some other presets showed promise in certain image slices, they were associated with limitations in others. However, we find that the combination of VMI and iMAR is beneficial for PCCT imaging in LVAD patients, corroborating the findings of Anhaus et al.^49^ In the case of the Hip preset, Anhaus et al.^49^ did not observe any qualitative or quantitative enhancement with energies exceeding 100 keV for hip implants. As for the spine, the optimal VMI was found to be at 100 keV. Our results align with these findings, indicating that 110 keV is the optimal choice. We found that the selection of conventional MAR did not improve the metal artifacts as much as the selection of kVp, keV, and kernel; the use of iMAR strongly reduced the metal artifacts, which agrees with Aissa et al.,^25^ Skornitzke et al.,^46^ and Anhaus et al.^49^

PCCT is expected to replace conventional CT partially and maybe even completely, initially for applications where it leads to major improvements in image quality. Our evaluation is a first step in this technological diagnostic leap for patients with LVADs, where it can bring benefits such as improved image quality to better device assessment and allows clinicians to accurately assess its position, integrity, and functioning at lower iodine contrast and radiation dose compared to conventional CT. Given that patients with LVADs are at an increased risk of kidney dysfunction, this capability could yield an important ability to conduct scans more safely in this patient group. Traditionally, flow monitoring and cardiac optimization for LVAD is performed with echocardiography. Our study shows that PCCT with optimal metal reduction settings, performed well in imaging and future retrospective images may be used to complement or replace echocardiography for regular scans and monitor changes. This longitudinal assessment facilitates tracking device performance, detect any evolving issues, and guide treatment adjustments.

One of the main limitations of this study was that the phantom was not anthropometric regarding iodine distribution, and the phantom images were not optimal due to the lungs not being fully expanded. However, as the location of the LVAD in relation to the heart muscle and blood pool has a large effect on the image quality, the use of biological materials (lamb) in combination with the chosen phantom setup is still considered to be the most optimal option to investigate the acquisition settings for a patient with LVAD. The effect of motion, the position and orientation of the LVAD, and the pump‐speed were not investigated in this study. Nevertheless, research conducted by Holmes et al.^22^ demonstrated that PCCT imaging retains spatial resolution and HU accuracy even when imaging objects in motion. Furthermore, only level 4 of QIR was evaluated. This was, however, previously shown to be the optimal reconstructions setting for coronary artery calcium scoring by Zsarnocazy et al.^19^ This in‐vivo study was limited to one patient, it is nevertheless the first time this patient group has been examined using PCCT, leading to valuable insights in the utility of PCCT within this patient population, contributing to the advancement of the field.

Reading images for follow‐up LVAD patients is a rather complex task, as it involves visualization of various tissues, also in proximity to large metal components. Consequently, radiologists may need to read several different reconstructions, as well as using different window levels and window width. We found two reconstructions methods that reduce metal artifacts and provide sufficient diagnostic interpretability. Nevertheless, it appears that the visibility of artifacts is primarily affected by keV and the reconstruction kernel, including the reconstruction matrix and how the raw data is collected and processed per pixel. Therefore, a better combination of MAR algorithms and reconstruction kernels may further improve the image quality. Ongoing research focuses on improving MAR algorithms, including the interpolation method and the utilization of both sinogram and image domains, as well as incorporating deep learning techniques.^50^ However, PCCT represents a technology that is new in the commercial market, and is therefore, as is the case with other new technologies, rapidly developing with a high rate of updates expected in the near future.

## 5 CONCLUSION

Our findings indicate that acquisition at 120 kVp with an IQ of 80, and subsequent reconstruction using either VMI 110 keV or T3D provides the highest image quality in patients with an LVAD. Optimal reconstruction kernels for visual assessment are Qr40f and Bv56f, while pacemaker iMAR preset should be employed. A slice thickness of 3.0 mm with overlap is advised for overall assessment. The volume of LVAD to measure blooming was shown to be the best objective method to assess metal artifacts, while the Fourier method for assessing streak artifacts was also shown to be effective. Overall, PCCT has the potential to revolutionize the evaluation of patients with left ventricular assist devices by offering improved image quality and thereby augmented device assessment.

## AUTHOR CONTRIBUTIONS

**Bente Konst**: Conceptualization; methodology; acquisition of phantom scans; data processing; statistical analysis; visualization; drafting of the manuscript. **Linus Ohlsson**: Methodology; phantom development; visual assessment of images; visualization; drafting of the manuscript. **Lilian Henriksson**: Protocol development and acquisition of patient scans; drafting of the manuscript. **Mårten Sandstedt**: Visual assessment of images; interpretation of results; drafting of the manuscript. **Anders Persson**: Conceptualization; methodology; visual assessment of images; critical revision of the manuscript. **Tino Ebbers**: Conceptualization; methodology; critical revision of the manuscript. All the authors reviewed the manuscript and approved the final submission.

## CONFLICT OF INTEREST STATEMENT

The authors have no conflict of interest to disclose.

## DATA AVAILABILTIY STATEMENT

The data that support the findings of this study are available from the corresponding author upon reasonable request.

## Supporting information

Supporting Information
