# Supplementary material for: Optimization of photon counting CT for cardiac imaging in patients with left ventricular assist devices: An in‐depth assessment of metal artifacts
Source: J Appl Clin Med Phys. 2024 May 13;25(7):e14386. doi: 10.1002/acm2.14386 (PMC11244676; doi:10.1002/acm2.14386)
Supplement: Supplementary file 1 — Supporting Information [file ACM2-25-e14386-s001.docx]

APPENDIX

| **TABLE A1.** Results from multinominal regression for the phantom. Score 1 is the reference and for non-ordered factors keV=40, no iMAR and kernel Qr36f were references. Numbers of regression coefficients with associated p-value less than 0.05 are marked with **bold**, otherwise they are marked with *italic*. Logg odd can be calculated according to Eq 1a. AIC=Akaike Information Criterion, relatively quality of the statistical model. The intercept gives the estimated log odds for the reference group Q36f, 40 keV, and no iMAR | | | | | | | | | | | | | | | | | | | |
| --- | --- | --- | --- | --- | --- | --- | --- | --- | --- | --- | --- | --- | --- | --- | --- | --- | --- | --- | --- |
|  | **e^β^ for Q1** | | | **e^β^ for Q2** | | | **e^β^ for Q3** | | | **e^β^ for Q4** | | | **e^β^ for Q5** | | | **e^β^ for Q6** | | | |
| **Response**  **Parameter** | **2** | **3** | **4** | **2** | **3** | **4** | **2** | **3** | **4** | **2** | **3** | **4** | **2** | **3** | **4** | **2** | **3** | **4** | **5** |
| **Intercept.** | *44.2* | **<0.001** | **<0.001** | *0.0864* | **<0.001** | **<0.001** | *35.6* | *22.9* | **<0.001** | **0.0813** | **<0.001** | **<0.001** | **0.00723** | **0.00089** | **<0.001** | *0.0116* | <0.001 | **0.0223** | **1.01** |
| **kVp** | *1.04* | **1.07** | **1.09** | *1.00* | **1.05** | *1.04* | *1.04* | *1.03* | *1.04* | **1.03** | *1.05* | *1.05* | *1.03* | **1.06** | **1.07** | *1.02* | 1.04 | *0.898* | **2.78** |
| **110 keV** | **110** | **>10 000** | **>10 000** | **7.47** | **4.92** | **>10 000** | **66.1** | **50.0** | **>10 000** | **13.3** | **>10 000** | **>10 000** | **40.7** | **261** | **166** | *17.3* | **>10 000** | **0.673** | *>10 000* |
| **190 keV** | **>10 000** | **>10 000** | **>10 000** | *7.78* | *1.61* | **>10 000** | **30.2** | *13.1* | **>10 000** | **10.8** | **0.0127** | **>10 000** | **>10 000** | **>10 000** | **>10 000** | *22.6* | <0.001 | **0.500** | **>10 000** |
| **62 keV** | **84** | **0.001** | **0.030** | *2.59* | *4.84* | **>10 000** | **22.6** | *6.55* | **0.0198** | **14.4** | **0.0270** | **0.00461** | **>10 000** | **>10 000** | **<0.001** | *11.3* | <0.001 | **0.0373** | **>10 000** |
| **67 keV** | *2.012* | **>10 000** | **<0.001** | *4.74* | *1.91* | **<0.001** | *3.64* | **<0.001** | **<0.001** | **3.73** | **<0.001** | **<0.001** | *4.95* | *4.17* | *3.85* | *2.45* | <0.001 | **0.439** | **11.6** |
| **70 keV SSP** | **517** | **>10 000** | **>10 000** | **<0.001** | **<0.001** | **>10 000** | **97.7** | **245** | **750** | **36.5** | **>10 000** | **>10 000** | **>10 000** | **>10 000** | **>10 000** | *14.0* | *3.63* | **>10 000** | **>10 000** |
| **T3D** | **57.1** | **>10 000** | **>10 000** | *3.10* | *5.94* | **>10 000** | **33.9** | **25.9** | **>10 000** | **11.1** | **>10 000** | **>10 000** | **53.3** | **451** | **130** | *15.0* | **>10 000** | **0.0160** | **>10 000** |
| **Extremities** | **>10 000** | **>10 000** | **>10 000** | *0.191* | *0.306* | **<0.001** | *0.507* | *1.22* | **<0.001** | **3.77** | **>10 000** | **<0.001** | *0.979* | *0.223* | *3.62* | *0.499* | <0.001 | **<0.001** | **1.65** |
| **Hip** | **>10 000** | **>10 000** | **>10 000** | *1.15* | *<0.001* | *0.649* | *0.380* | *3.66* | **>10 000** | **3.77** | *>10 000* | *7.94* | *0.979* | *0.223* | *2.42* | *1.00* | <0.001 | **v** | **2.71** |
| **Pacemaker** | **8.57** | **25.3** | **>10 000** | *2.43* | *7.35* | *2.01* | *4.24* | **19.9** | **>10 000** | **1.76** | *>10 000* | *0.253* | *2.56* | *1.45* | *4.60* | *1.13* | *>10 000* | **3.01** | **3.08** |
| **Thoracal** | *0.184* | *1.553* | **<0.001** | *0.0717* | *0.229* | **<0.001** | *0.253* | *2.44* | **<0.001** | **<0.001** | **<0.001** | **<0.001** | *0.489* | **<0.001** | **<0.001** | *0.166* | <0.001 | **<0.001** | **1.18** |
| **Bl56f** | **<0.001** | **<0.001** | **<0.001** | **<0.001** | **<0.001** | **<0.001** | **<0.001** | **<0.001** | **<0.001** | *0.444* | **0.667** | **<0.001** | **<0.001** | **<0.001** | **<0.001** | *0.167* | *>10 000* | **0.392** | **1.18** |
| **Bv36f** | **46.3** | **139** | **15.435** | **<0.001** | **<0.001** | **<0.001** | **>10 000** | **>10 000** | **>10 000** | *1.00* | **1.00** | **0.00271** | **<0.001** | **<0.001** | **<0.001** | *1.33* | <0.001 | **2.43** | **3.79** |
| **Bv40f** | **<0.001** | **<0.001** | **<0.001** | **<0.001** | **<0.001** | **<0.001** | **<0.001** | **<0.001** | **<0.001** | *0.931* | **2.07** | **>10 000** | **<0.001** | **<0.001** | **<0.001** | *1.13* | *>10 000* | **0.122** | **3.09** |
| **Bv44f** | **>10 000** | **>10 000** | **<0.001** | **<0.001** | **<0.001** | **<0.001** | **<0.001** | **<0.001** | **<0.001** | *1.00* | **1.00** | **0.00271** | **<0.001** | **<0.001** | **<0.001** | *1.33* | <0.001 | **2.43** | **3.79** |
| **Bv56f** | **66.7** | **88.9** | **14.8** | **<0.001** | **<0.001** | **<0.001** | **<0.001** | **<0.001** | **<0.001** | **0.667** | **<0.001** | **<0.001** | **<0.001** | **<0.001** | **<0.001** | *0.222* | <0.001 | **0.098** | **1.25** |
| **Qr40f** | **<0.001** | **<0.001** | **<0.001** | **<0.001** | **<0.001** | **<0.001** | **<0.001** | **<0.001** | **<0.001** | *1.24* | **1.65** | **>10 000** | **<0.001** | **<0.001** | **<0.001** | *0.763* | *>10 000* | **0.101** | **2.15** |
| **Qr44f** | **55.5** | **37.0** | **24.7** | **<0.001** | **<0.001** | **<0.001** | **<0.001** | **<0.001** | **<0.001** | *1.00* | **1.00** | **0.00271** | **<0.001** | **<0.001** | **<0.001** | *1.00* | <0.001 | **2.43** | **2.72** |
| **slice** | *1.28* | *1.80* | *2.48* | **>10 000** | **>10 000** | **>10 000** | *1.85* | *2.58* | *2.98* | *1.38* | *1.74* | *0.833* | **>10 000** | **>10 000** | **>10 000** |  |  |  |  |
| **IQ** |  |  |  |  |  |  |  |  |  | *0.948* | *1.02* | *0.908* | *0.979* | *0.956* | *1.00* |  |  |  |  |
| **AIC** | 570 | | | 617 | | | 608 | | | 551 | | | 582 | | | 648 | | | |
| **Deviance** | 450 | | | 497 | | | 488 | | | 425 | | | 456 | | | 496 | | | |

| **TABLE A2.** Results from multinominal regression for the patient. Score (grading) 1 is the reference and for non ordered factors keV=40, non iMAR and kernel Qr36f were references. Numbers of regression coefficients with associated p-value less than 0.05 are marked with **bold**, otherwise they are marked with *italic*. Logg odd can be calculated according to Eq 1a. The intercept gives the estimated log odds for the reference group Q36f, 40 keV, and no iMAR | | | | | | | | | | | | | | |
| --- | --- | --- | --- | --- | --- | --- | --- | --- | --- | --- | --- | --- | --- | --- |
|  | **e^β^ for Q1** | | **e^β^ for Q2** | | **e^β^ for Q3** | | **e^β^ for Q4** | | **e^β^ for Q5** | | | **e^β^ for Q6** | | |
| **Score**  **Parameter** | **2** | **3** | **2** | **3** | **2** | **3** | **2** | **3** | **2** | **3** | **4** | **2** | **3** | **4** |
| **Intercept** | **<0.001** | **<0.001** | **<0.001** | **<0.001** | *0.238* | **<0.001** | *0.531* | **<0.001** | **<0.001** | **<0.001** | **<0.001** | *0.385* | **<0.001** | **<0.001** |
| **110 keV** | **>10 000** | **>10 000** | **>10 000** | **19.1** | *1.21* | **>10 000** | *1.65* | **>10 000** | **>10 000** | **>10 000** | **5256** | *5.70* | **>10 000** | **4.61** |
| **190 keV** | **>10 000** | **<0.001** | **>10 000** | **0.00152** | *1.00* | **<0.001** | *4.00* | **<0.001** | *4.50* | *6.00* | **0.00118** | *6.00* | **>10 000** | **1.42** |
| **62 keV** | **>10 000** | **<0.001** | **>10 000** | **<0.001** | *1.00* | **<0.001** | *2.00* | **<0.001** | *7.50* | **<0.001** | **0.207** | *2.00* | **<0.001** | **0.0294** |
| **90 keV** | **>10 000** | **0.002499** | **>10 000** | **0.161** | *1.81* | **<0.001** | *5.69* | **<0.001** | **>10 000** | **>10 000** | **<0.001** | *6.35* | **>10 000** | **<0.001** |
| **70 keV SSP** | **>10 000** | **0.001981** | **>10 000** | **0.000258** | *2.00* | **<0.001** | *4.00* | **<0.001** | **>10 000** | **>10 000** | **5.60** | *10.0* | **<0.001** | **7.28** |
| **T3D** | **>10 000** | **>10 000** | **>10 000** | **>10 000** | *2.32* | **>10 000** | *5.01* | **>10 000** | **>10 000** | **>10 000** | **8677** | *2.54* | **>10 000** | **11.8** |
| **Extremities** | *20.0* | **>10 000** | *4.00* | **0.00569** | *7.50* | **>10 000** | *0.667* | **>10 000** | **>10 000** | **>10 000** | **19.3** | *0.999* | **>10 000** | **>10 000** |
| **Hip** | *3.33* | **>10 000** | *4.00* | **0.00569** | *3.33* | **>10 000** | *1.50* | **>10 000** | **>10 000** | **>10 000** | **19.3** | *0.500* | **>10 000** | **0.0336** |
| **Pacemaker** | *2.62* | **>10 000** | *1.49* | **0.147** | *3.03* | **>10 000** | *0.827* | **>10 000** | **>10 000** | **>10 000** | **0.00547** | *1.43* | **>10 000** | **0.0462** |
| **Thoracal** | *0.608* | **>10 000** | *0.444* | **>10 000** | *3.58* | **>10 000** | *2.22* | **>10 000** | **>10 000** | **>10 000** | **<0.001** | *2.74* | **>10 000** | **<0.001** |
| **Bl56f** | *0.500* | *2.00* |  |  | *1.00* | **<0.001** | *2.00* | **0.001538** | **<0.001** | **<0.001** | **<0.001** | *1.00* | *1.00* | **0.192** |
| **Bv36f** | *0.750* | **<0.001** |  |  | *0.500* | **<0.001** | *2.00* | **0.001538** | **>10 000** | **>10 000** | **12.2** | *1.33* | *0.500* | **0.193** |
| **Bv40f** | *0.750* | **<0.001** |  |  | *1.00* | **<0.001** | *2.00* | **0.001538** | **>10 000** | **>10 000** | **17.8** | *1.00* | *1.00* | **0.192** |
| **Bv44f** | *1.50* | **<0.001** |  |  | *1.00* | **<0.001** | *1.00* | **<0.001** | **<0.001** | **<0.001** | **<0.001** | *1.00* | *1.00* | **0.192** |
| **Bv56f** | *0.515* | *0.141* |  |  | *0.374* | **>10 000** | *0.920* | **>10 000** | **<0.001** | **<0.001** | **>10 000** | *0.536* | *0.360* | **>10 000** |
| **Qr40f** | *0.787* | *0.190* |  |  | *0.605* | **>10 000** | *0.827* | **>10 000** | *2.90* | *1.43* | **0.0446** | *0.951* | *0.660* | **<0.001** |
| **Qr44f** | *0.375* | *0.750* |  |  | *1.000* | **<0.001** | *1.00* | **<0.001** | **<0.001** | **<0.001** | **<0.001** | *1.33* | *0.500* | **0.193** |
| **Qr56f** | *0.750* | **<0.001** |  |  | *0.200* | **<0.001** | *1.00* | **<0.001** | **<0.001** | **<0.001** | **<0.001** | *0.333* | *0.500* | **0.00526** |
| **slice** | *1.59* | *1.72* | *0.871* | *1.56* | *1.42* | *1.42* | **2.22** | **3.86** | *1.01* | *1.60* | *1.70* | *0.897* | *1.03* | *1.59* |
| **AIC** | 323 | | 287 | | 330 | | 323 | | 392 | | | 438 | | |
| **Deviance** | 243 | | 207 | | 250 | | 243 | | 272 | | | 318 | | |

| **TABLE A3**. The regression coefficients for the models given in Table 5 for the difference in HU between tissue with metal artifact and normal tissue, and for the standard deviation for tissue with and without metal artifact for the phantom and the patient images. | | | | | | | | |
| --- | --- | --- | --- | --- | --- | --- | --- | --- |
|  | **Diff_HU_** | | | | **SD_ARTIFACT_** | | | |
|  | **Phantom** | | **Patient** | | **Phantom** | | **Patient** | |
| **β** | **Estimate** | **p** | **Estimate** | **p** | **Estimate** | **p** | **Estimate** | **p** |
| **Intercept** | 52.6 | 0.647 | 5.65 | < 0.001 | 11.2 | < 0.001 | 5.09 | < 0.001  - |
| **kvp/log(kvp)** | -0.648 | 0.356 | - | - | -0.894 | 0.00659 | - |  |
| **110 keV** | 93.7 | 0.0163 | -1.03 | < 0.001 | -0.695 | < 0.001 | -0.894 | < 0.001 |
| **190 keV** | 76.1 | 0.189 | -0.937 | 0.00189 | -0.481 | 0.0240 | -0.851 | < 0.001 |
| **62 keV** | 98.1 | 0.096 | -0.673 | 0.00638 | -0.646 | 0.00433 | -0.435 | 0.00205 |
| **90 keV** | - | - | -0.924 | 0.00199 | - | - | -1.16 | < 0.001 |
| **67 keV** | 56.0 | 0.326 | - | - | -0.443 | 0.0352 | - | - |
| **70 keV** | -144 | 0.077 | -0.554 | 0.0126 | -0.624 | 0.0347 | -0.504 | 0.00118 |
| **T3D keV** | 34.9 | 0.409 | -0.852 | 0.00269 | -0.533 | 0.00214 | -0.916 | < 0.001 |
| **IQ/log(IQ)** | - | - | - | - | -0.275 | 0.302 | - | - |
| **slice/log(slice)** | -16.1 | 0.249 | -0.030 | 0.498 | -0.136 | 0.0405 | -0.00179 | 0.931 |
| **iMAR Extremities** | 127 | 0.0632 | 1.58 | < 0.001 | -0.936 | 0.000745 | 1.05 | < 0.001 |
| **iMAR Hip** | 147 | 0.0347 | 1.53 | < 0.001 | -0.652 | 0.00971 | 1.07 | < 0.001 |
| **iMAR Pacemaker** | -195 | 0.00184 | 1.17 | < 0.001 | -0.341 | 0.0758 | 0.722 | < 0.001 |
| **iMAR Thoracal** | 82.0 | 0.215 | 0.898 | 0.00222 | 0.097 | 0.664 | 0.926 | < 0.001 |
| **Kernel Bl56f** | 88.0 | 0.185 | 0.0461 | 0.738 | 0.165 | 0.463 | -0.295 | 0.00847 |
| **Kernel Bv36f** | 18.0 | 0.780 | 0.0319 | 0.816 | 0.0782 | 0.725 | -0.022 | 0.735 |
| **Kernel Bv40f** | -253 | 0.018 | -0.170 | 0.257 | -0.245 | 0.472 | -0.858 | < 0.001 |
| **Kernel Bv44f** | 30.0 | 0.642 | 0.00295 | 0.983 | -0.1301 | 0.560 | -0.665 | < 0.001 |
| **Kernel Bv56f** | 34.0 | 0.599 | -0.967 | 0.0169 | 0.115 | 0.606 | -0.837 | 0.00199 |
| **Kernel Qr40f** | 42.6 | 0.377 | -0.199 | 0.151 | 0.0364 | 0.827 | -0.583 | < 0.001 |
| **Kernel Qr44f** | 1.00 | 0.988 | 0.0601 | 0.665 | 0.0241 | 0.914 | -0.604 | < 0.001 |
| **Kernel Qr56f** | - | - | 0.158 | 0.287 | - | - | -0.276 | 0.0107 |
| **iMARPa:kernelBv40f** | 305 | 0.00105 | - | - | 0.220 | 0.412 | - | - |
| **keV110:kernelBv40f** | -21.8 | 0.730 | - | - | -0.0567 | 0.797 | - | - |
| **keV70:kernelBv40f** | 289 | 0.0148 | - | - | 0.251 | 0.498 | - | - |
| **iMARPa:kernelBv56f** | - | - | 0.810 | 0.00548 | - | - | 0.561 | 0.00135 |
| **keV110:kernelBv56f** | - | - | 0.260 | 0.147 | - | - | -0.125 | 0.144 |
| **keV90:kernelBv56f** | - | - | 0.287 | 0.184 | - | - | 0.265 | 0.0356 |
| Residual standard errror | 44.8 |  | 0.0909 |  | 0.154 |  | 0.0432 |  |

| **TABLE A4**. The regression coefficients for the models given in Table 6 for the measured quantities of AmplitudeLowFreq and BloomVol for the phantom and the patient images. | | | | | | | | |
| --- | --- | --- | --- | --- | --- | --- | --- | --- |
|  | **AmplitudeLowFreq** | | | | **BloomVol** | | | |
|  | **Phantom** | | **Patient** | | **Phantom** | | **Patient** | |
| **β** | **Estimate** | **p** | **Estimate** | **p** | **Estimate** | **p** | **Estimate** | **p** |
| **Intercept** | 18293 | 0.0456 | 90442 | < 0.001 | 12.6 | < 0.001 | 11.3 | < 0.001 |
| **kvp/log(kvp)** | 174 | 0.0109 | - | - | -0.221 | < 0.001 | - | - |
| **110 keV** | 5241 | 0.110 | -50022 | < 0.001 | -0.426 | < 0.001 | -0.817 | < 0.001 |
| **190 keV** | 7675 | 0.154 | -33623 | 0.00162 | -0.568 | < 0.001 | -1.00 | < 0.001 |
| **62 keV** | 82 | 0.0456 | -32213 | 0.00176 | -0.0923 | < 0.001 | -0.270 | < 0.001 |
| **90 keV** | - | - | -43269 | < 0.001 | - | - | -0.682 | < 0.001 |
| **67 keV** | 3068 | 0.988 | - | - | -0.0961 | < 0.001 | - | - |
| **70 keV** | -1057 | 0.563 | -28517 | 0.00225 | -0.210 | < 0.001 | -0.432 | < 0.001 |
| **T3D keV** | 9653 | 0.811 | -46323 | < 0.001 | -0.166 | < 0.001 | -0.523 | < 0.001 |
| **slice/log(slice)** | 1507 | 0.012 | 1701 | 0.211 | 0.0124 | 0.0671 | -0.194 | 0.951 |
| **iMAR Extremities** | - | - | -19888 | 0.00461 | -0.0324 | 0.156 | -0.00245 | 0.945 |
| **iMAR Hip** | - | - | -20430 | 0.00437 | -0.0400 | 0.084 | -0.00272 | 0.769 |
| **iMAR Pacemaker** | - | - | -18966 | 0.00416 | -0.0415 | 0.00645 | -0.00967 | 0.794 |
| **iMAR Thoracal** | - | - | -21763 | 0.00385 | -0.0317 | 0.165 | -0.00927 | 0.00586 |
| **Kernel Bl56f** | - | - | 30752 | 0.00194 | -0.359 | < 0.001 | -0.150 | 0.839 |
| **Kernel Bv36f** | - | - | 1709 | 0.334 | 0.0337 | 0.179 | 0.00809 | 0.938 |
| **Kernel Bv40f** | - | - | 388 | 0.802 | 0.00702 | 0.721 | -0.00309 | 0.798 |
| **Kernel Bv44f** | - | - | 18525 | 0.00531 | 0.00908 | 0.711 | -0.0102 | 0.137 |
| **Kernel Bv56f** | - | - | 28943 | 0.00868 | -0.0794 | 0.00425 | -0.0550 | 0.626 |
| **Kernel Qr40f** | - | - | -2678 | 0.161 | -0.000804 | 0.964 | -0.0162 | 0.625 |
| **Kernel Qr44f** | - | - | 10888 | 0.0151 | -0.0357 | 0.157 | -0.0196 | 0.104 |
| **Kernel Qr56f** | - | - | 19727 | 0.00468 | - | - | - | - |
| **kev110:slice** | - | - | -2659 | 0.0784 | - | - | - | - |
| **kev110:kernelBv56f** | - | - | 12432 | 0.0213 | - | - | - | - |
| **kev90:kernelBv56f** | - | - | 6290 | 0.0892 | - | - | - | - |
| **slice:kernelBv56f** | - | - | -4741 | 0.0213 | - | - | - | - |
| **kernelBv56f:iMARPa** | - | - | -16528 | 0.0108 | - | - | - | - |
| Residual standard errror | 4248 |  | 958 |  | 0.0170 |  | 0.0271 |  |
